# ENPP1 and IFIT2 in PBMCs as early predictive biomarkers for HBsAg clearance and responses to Peg-IFN-α in HBeAg-negative chronic hepatitis B patients

**DOI:** 10.3389/fimmu.2026.1796228

**Published:** 2026-06-10

**Authors:** Hao Pang, Xinglin Fu, Jinhong Jiang, Bo Qin

**Affiliations:** 1 Department of Infectious Diseases, Chongqing Key Laboratory of Infectious Diseases and Parasitic Diseases, The First Affiliated Hospital of Chongqing Medical University, Chongqing, China; 2 Central Laboratory, The First Affiliated Hospital of Chongqing Medical University, Chongqing, China

**Keywords:** ENPP1, hepatitis B virus, IFIT2, Peg-IFN-α, response

## Abstract

**Objective:**

To investigate whether early on-treatment expression of ectonucleotide pyrophosphatase/phosphodiesterase 1 (ENPP1) and interferon-induced protein with tetratricopeptide repeats 2 (IFIT2) in peripheral blood mononuclear cells (PBMCs) could serve as biomarkers for predicting hepatitis B surface antigen (HBsAg) clearance and therapeutic responses to pegylated interferon-α (Peg-IFN-α) in HBeAg-negative chronic hepatitis B (CHB) patients.

**Methods:**

In this prospective study, HBeAg-negative CHB patients received Peg-IFN-α therapy for 48 weeks. Patients were categorized into virological response (VR) or non-virological response (NVR) groups based on changes in HBV DNA and HBsAg levels at week 48, and further stratified into serological response (SR) or non-serological response (NSR) groups based on the occurrence of HBsAg loss or seroconversion. ENPP1 and IFIT2 mRNA levels in PBMCs were quantified using real-time quantitative PCR. Correlations between gene expression and HBsAg reduction were evaluated using Spearman’s rank correlation analysis. Factors independently associated with VR and SR were identified using univariate and multivariate logistic regression analyses. The discriminative ability of individual biomarkers and combined predictive models was assessed by receiver operating characteristic (ROC) curve analysis.

**Results:**

Both ENPP1 and IFIT2 mRNA levels were significantly elevated in the VR and SR groups during early treatment. Expression levels of both genes at weeks 12 and 24 positively correlated with the magnitude of HBsAg reduction, and their early induction at week 12 was associated with subsequent HBsAg decline. Multivariate analyses identified ENPP1 and IFIT2 mRNA levels at weeks 12 and 24 as independent predictors of VR and SR. ENPP1 expression at week 12 demonstrated the highest predictive accuracy for VR (AUC = 0.7645), while ENPP1 at week 24 showed moderate predictive value for SR (AUC = 0.7399). In contrast, IFIT2 consistently exhibited superior predictive performance for VR (AUC = 0.8791) and for SR at week 24 (AUC = 0.8879). The combination of ENPP1 and IFIT2 further improved predictive accuracy for both VR (AUC = 0.9098) and SR (AUC = 0.9217).

**Conclusions:**

ENPP1 and IFIT2 in PBMCs represent valuable biomarkers for early prediction of Peg-IFN-α treatment efficacy and HBsAg clearance in HBeAg-negative CHB patients.

## Introduction

Hepatitis B virus (HBV) infection continues to impose a substantial global disease burden. According to estimates from the World Health Organization (WHO), the prevalence of chronic infection reached 254 million individuals in 2022, resulting in approximately 1.1 million deaths annually from liver-related complications such as cirrhosis and hepatocellular carcinoma (HCC) (1, 2). Despite the availability of prophylactic vaccines, HBV continues to drive a considerable number of new infections, with 1.2 million new cases reported annually (3). Although potent antiviral agents are now widely used, the long-term clinical control of chronic hepatitis B (CHB) remains suboptimal. In routine practice, currently available therapies rarely result in durable viral control or functional cure, which is defined by sustained hepatitis B surface antigen (HBsAg) loss, irrespective of anti-HBs seroconversion. These persistent therapeutic constraints highlight the continuing need for more effective and dependable treatment approaches.

Evidence suggests that an estimated approximately 10% of individuals in the immune-tolerant phase convert spontaneously to the HBeAg-negative chronic HBV infection stage annually (4). While this phase is typically characterized by mild hepatic inflammation and the absence of cirrhotic progression (5, 6), and thus antiviral therapy is not routinely recommended by current guidelines (7), clinical stability is not guaranteed. Emerging evidence suggests that a subset of HBeAg-negative patients may experience disease reactivation driven by viral mutations and immune escape, significantly increasing the risk of advanced liver complications (8). Moreover, HBeAg-negative CHB is associated with an elevated risk of mortality and HCC compared with the general population (9, 10). This heterogeneity underscores the potential value of interferon therapy in selected HBeAg-negative patients exhibiting indicators of immune activity. These observations underscore the limitations of current risk stratification strategies and emphasize the necessity for personalized therapeutic decision-making.

At present, the clinical management of CHB primarily relies on pegylated interferon-α (Peg-IFN-α) and nucleos(t)ide analogues (NUCs). Although NUCs effectively suppress HBV replication, they rarely achieve a functional cure (11), necessitating indefinite medication due to the high risk of viral relapse upon discontinuation (12, 13). In contrast, Peg-IFN-α offers a finite duration of treatment and achieves higher rates of serological response (14). However, its clinical utility is hampered by substantial adverse effects and marked interindividual variability in treatment response, with only a minority of patients achieving functional cure. Therefore, identifying reliable biomarkers to predict therapeutic response and guide patient selection remains a critical unmet need (15). Although quantitative HBsAg levels and on-treatment declines have shown some predictive value, they often lack sufficient specificity, prompting the search for novel host immune-related biomarkers (16, 17).

Elucidating the host determinants governing these immune responses is essential for developing predictive tools. Mechanistically, Peg-IFN-α exerts its antiviral activity primarily through the induction of interferon-stimulated genes (ISGs). Upon receptor ligation, the Janus kinase/signal transducer and activator of transcription (JAK/STAT) pathway is activated, resulting in the transcriptional upregulation of diverse ISGs that orchestrate a potent antiviral state (18, 19). Emerging evidence has linked specific ISG expression patterns to treatment outcomes in Peg-IFN-α–treated CHB patients (20). For instance, ISGs such as TRIM25, MX2, IFI27, and MyD88 play pivotal roles in host immunity and HBV suppression (21–24). Despite these advances, clinically validated ISG-based biomarkers for predicting Peg-IFN-α response remain limited, and the functional relevance of specific ISGs in treatment outcomes warrants further investigation. Among potentially predictive ISGs, ectonucleotide pyrophosphatase/phosphodiesterase 1 (ENPP1) and interferon-induced protein with tetratricopeptide repeats 2 (IFIT2) have recently emerged as promising candidates due to their direct involvement in antiviral and immune regulatory pathways.

ENPP1, belonging to the ecto-nucleotide pyrophosphatase/phosphodiesterase enzyme family, is characterized structurally as a type II transmembrane glycoprotein and is localized to both the plasma membrane and the lumen of the endoplasmic reticulum (25). Initially identified through high-throughput screening and subsequently established as an ISG (26), ENPP1 has been reported to inhibit HBV replication by suppressing the transcriptional activity of the HBV pregenomic promoter, thereby downregulating pregenomic RNA (pgRNA) and hepatitis B core antigen (HBcAg) levels (27). Similarly, the IFIT family comprises highly expressed ISGs with RNA-binding capabilities critical for antiviral defense (28). As a key member of this family, IFIT2 has been demonstrated to suppress viral replication by inhibiting HBV SP1 and SP2 promoter activity (29–31). However, despite these mechanistic insights from preclinical models, the clinical relevance of ENPP1 and IFIT2 as predictive biomarkers for Peg-IFN-α therapeutic response and HBsAg clearance, particularly in HBeAg-negative CHB patients, has not been systematically evaluated. Given that these factors directly modulate HBV transcription, we hypothesized that their expression levels could serve as critical determinants of therapeutic efficacy.

Therefore, the present study aimed to investigate the associations between ENPP1 and IFIT2 mRNA expression levels and Peg-IFN-α treatment response, as well as their relationship with HBsAg clearance. By identifying ISG-based predictive biomarkers, this study seeks to provide a rationale for personalized Peg-IFN-α treatment and improved patient stratification in clinical practice.

## Materials and methods

### Patient cohort and study design

This prospective cohort study initially screened 94 HBeAg-negative patients with chronic HBV infection at the Department of Infectious Diseases, The First Affiliated Hospital of Chongqing Medical University, between October 2023 and October 2024. Diagnosis and clinical management were performed in accordance with the 2022 guidelines of the Chinese Society of Hepatology and the Chinese Society of Infectious Diseases (7). Inclusion criteria were as follows: (1) aged 18–65 years; (2) HBsAg positivity and HBeAg negativity with detectable anti-HBe and anti-HBc; (3) HBV DNA levels < 2,000 IU/mL; (4) normal hepatic biochemical indices; and (5) histological or clinical evidence of mild or no liver inflammation or fibrosis. Exclusion criteria included: (1) co-infection with other viruses (HCV, HDV, HIV, or EBV); (2) concomitant liver diseases such as autoimmune hepatitis, alcoholic liver disease, or metabolic-associated fatty liver disease (MAFLD); (3) history of malignancy; or (4) any contraindications to interferon therapy.

After exclusion of 3 patients who did not meet the inclusion criteria, 91 patients were prospectively enrolled and received pegylated interferon alfa-2b (Peg-IFN-α2b) therapy at a standard dose of 180 μg administered subcutaneously once weekly for 48 weeks. Concomitant use of other antiviral agents or immunomodulatory therapies was not permitted during the study period. During follow-up, 2 patients were lost to follow-up and 1 patient did not complete scheduled examinations, resulting in incomplete longitudinal data. Consequently, 88 patients were included in the final per-protocol analysis. Patients with incomplete longitudinal follow-up data were excluded from the final longitudinal analyses, and no missing- data imputation was performed. Common adverse events, including influenza-like symptoms, leukopenia, and thrombocytopenia, were monitored throughout treatment and were manageable with standard supportive care. No patients permanently discontinued Peg-IFN-α2b therapy because of adverse events. The study design is illustrated in **Supplementary Figure 1**. Furthermore, to further evaluate the robustness and generalizability of the established models, an independent external validation cohort comprising 53 HBeAg-negative CHB patients was enrolled by April 2025 and completed a 48-week Peg-IFN-α treatment course between April 2025 and April 2026. This cohort was completely separate from the training cohort with no patient overlap, and all participants met the identical inclusion and exclusion criteria. In addition, 44 untreated CHB patients and 37 healthy controls (HCs) were recruited for baseline comparisons. The study protocol was approved by the Ethics Committee of The First Affiliated Hospital of Chongqing Medical University (Approval No. 2023-311).

### Study procedures

Serum HBsAg was monitored at 12-week intervals. Venous blood samples were collected at weeks 0, 12, and 24 for PBMC isolation and subsequent quantification of ENPP1 and IFIT2 mRNA expression.

### Study endpoints

Endpoints at week 48 were defined using modified criteria to reflect clinically meaningful changes in this low-viremia population. Accordingly, treatment outcomes were categorized as follows: (1) Virological response (VR): defined as an HBV DNA decline > 1 log10 IU/mL or a significant HBsAg decline (> 1 log10 IU/mL or HBsAg clearance). Patients not meeting these criteria were classified into the non-virological response (NVR) group. (2) Serological response (SR): defined as HBsAg loss or seroconversion. Patients not achieving this endpoint were classified as the non-serological response (NSR) group.

### Clinical and laboratory measurements

Serum HBV DNA loads were quantified via fluorescent real-time PCR using the Cobas Z480 system (Roche, Switzerland), with a lower limit of detection of 50 IU/mL. Serological markers, including HBsAg, anti-HBs, HBeAg, and anti-HBe, were analyzed using the Abbott Architect i2000 automated immunoassay system. Liver function tests were performed on an automated chemistry analyzer (Cobas, Roche, Shanghai), and routine hematological parameters were processed using the Mindray BC-6600 hematology analyzer (Shanghai). Additionally, HBV genotype was determined during sample collection using standard clinical testing procedures. In this cohort, only genotypes B and C were identified, which are the predominant genotypes in this region.

### RNA extraction and quantitative real-time PCR

Total RNA was isolated from PBMCs using TRIzol reagent (Invitrogen, Carlsbad, CA, USA) and subsequently reverse transcribed into cDNA using a cDNA synthesis kit. Quantitative real-time PCR (qRT-PCR) for ENPP1 and IFIT2 was performed on a Bio-Rad CFX96 system (Bio-Rad, USA). The amplification program consisted of an initial denaturation step at 95 °C for 30 s, followed by 40 cycles at 95 °C for 5 s and 60 °C for 30 s. Melt curve analysis was carried out immediately after amplification to verify product specificity, as described in the **Supplementary Methods**. Gene expression was quantified using a relative approach, with GAPDH serving as the internal reference. Primer sequences employed for qRT-PCR are provided in **Supplementary Table 1**. Relative mRNA expression levels of ENPP1 and IFIT2 were calculated using the comparative cycle threshold (2^−ΔΔCt) method (32). In accordance with MIQE guidelines (33), primer amplification efficiencies for ENPP1, IFIT2, and GAPDH were verified using standard curve analysis based on serial dilutions of cDNA, yielding efficiencies between 90% and 110% with excellent linearity (R² > 0.99). The stability of GAPDH as the internal reference gene was systematically validated; as shown in **Supplementary Figure 2**, no statistically significant variation in raw Ct values was observed across different study groups, clinical response groups, or longitudinal treatment time points (all P > 0.05). All qRT-PCR reactions were performed in technical triplicates, and mean Ct values were used for downstream analysis. Intra-assay and inter-assay coefficients of variation (CVs) were <1.5% and <5%, respectively. Reactions exhibiting a standard deviation >0.5 Ct among triplicates were repeated to ensure data reliability.

### Specification of combined predictive models

Combined predictive models were constructed using multivariable logistic regression. For VR prediction, a sequential model incorporating ENPP1 mRNA at week 12 and IFIT2 mRNA at week 24 was developed. For SR prediction, a dual-marker model including both ENPP1 and IFIT2 mRNA at week 24 was established. The model for VR can be expressed as: logit(PVR) = β_0_ + β_1_ × ENPP1week12 + β_2_ × IFIT2week24. The model for SR can be expressed as: logit(PSR) = β_0_ + β_1_ × ENPP1week24 + β_2_ × IFIT2week24. Predicted probabilities were derived from the fitted logistic regression equations. The optimal probability thresholds derived from the training cohort using the Youden index were subsequently applied to the internal and external validation cohorts. Detailed model parameters, including intercepts (β_0_), coefficients (β_1_, β_2_) with 95% confidence intervals, optimal probability thresholds, and performance metrics, are provided in **Supplementary Table 2**.

### Statistical analysis

All statistical analyses and graphical representations were executed using SPSS version 27.0 and GraphPad Prism version 10.1.2, and R software version 4.5.3. Prior to statistical testing, serum HBsAg and HBV DNA values underwent logarithmic transformation. Quantitative data are presented as mean ± standard deviation (SD) for normally distributed variables, and as median with interquartile range (IQR) for non-normally distributed variables. Longitudinal changes were analyzed using repeated-measures ANOVA (parametric) or the Friedman test (non-parametric). Between-group comparisons were performed using the Student’s t-test or the Mann–Whitney U test, as appropriate. Correlations were assessed using Pearson’s correlation coefficient for normally distributed data and Spearman’s rank correlation for non-normally distributed data. To evaluate sample size adequacy for multivariable modeling and minimize the risk of overfitting, the events per variable (EPV) principle was applied. Based on the number of observed clinical endpoints in the training cohort, the final multivariable models were intentionally restricted to a maximum of three predictors, consistent with the recommended minimum EPV threshold of 10. Univariate logistic regression analyses were first performed to identify variables potentially associated with VR and SR. Candidate variables for multivariable modeling included clinically relevant covariates together with variables showing P < 0.10 in univariate analyses. Final multivariable logistic regression models were then constructed using forward stepwise selection while maintaining the prespecified EPV constraint. The predictive performance of these models was evaluated based on the area under the receiver operating characteristic curve (AUC), and the DeLong test was used to compare differences between AUC values. The incremental predictive performance of the combined model over conventional markers was further assessed using continuous net reclassification improvement (NRI) and integrated discrimination improvement (IDI), with 95% confidence intervals obtained via bootstrap resampling. To ensure the robustness and generalizability of the established models, both internal and external validations were performed. Internal validation was conducted in the training cohort using bootstrap resampling (1,000 iterations) to assess model stability and estimate optimism-corrected performance. Model discrimination was quantified using the concordance index (C-index), while calibration was assessed via calibration curves, intercept, slope, and Brier score. For external validation, the predictive models developed in the training cohort were directly applied to the independent validation cohort without any updating or recalibration. The predefined cut-off values were strictly preserved to ensure an unbiased assessment. In this cohort, model discrimination was evaluated using the AUC, and calibration performance was assessed similarly using calibration curves, intercept, slope, and Brier score. Finally, clinical utility for both internal and external validations was evaluated using decision curve analysis (DCA). A two-tailed P-value of < 0.05 was considered statistically significant.

## Results

### Baseline characteristics and treatment responses in HBeAg-negative CHB patients receiving Peg-IFN-α treatment

The baseline demographic and clinical data of the 88 Peg-IFN-α-treated patients, 44 untreated CHB patients, and 37 healthy controls are provided in **Table 1**. Except for baseline serum HBsAg levels, which were significantly lower in the treatment group, no significant differences were observed regarding age, gender, HBV DNA, ALT, AST, platelets (PLT), or white blood cells (WBC). Additionally, an independent external validation cohort comprising 53 HBeAg-negative CHB patients was enrolled. The baseline characteristics of the validation cohort were comparable to those of the training cohort, with no significant differences observed in age, gender, HBsAg, HBV DNA, AST, ALT, WBC, or PLT (**Supplementary Table 3**). Upon completion of the 48-week regimen, 39 patients (44.32%) attained a virological response (VR), and 31 (35.23%) achieved serological response (SR) (see **Supplementary Tables 4**, **S5**).

**Table 1:** Baseline characteristics in the groups.

Characteristics	HC	Untreated CHB	CHB treated with Peg-IFN-α	P value
Number(n)	37	44	88	
Age(year)	33.11 ± 8.701	39.75 ± 10.67	45.00(35.00,51.00)	0.1319
Gender(male/female)	19/18	23/21	41/47	
HBsAg (log10 IU/mL)	UD	3.109(2.944,3.376)	2.846(1.707,3.358)	**0.0232**
HBV DNA (log10 IU/mL)	UD	1.699(1.699,2.691)	1.699(1.699,3.000)	0.7994
AST(U/L)	21.00(17.00,27.00)	24.00(20.25,31.75)	24.00(21.25,31.00)	0.8520
ALT(U/L)	25.27 ± 7.370	19.00(14.00,37.50)	25.50(17.25,39.00)	0.0867
WBC(×10^9/L)	5.620(4.410,6.885)	4.198 ± 1.289	4.348 ± 1.268	0.5293
PLT(×10^9/L)	198.8 ± 51.81	162.5 ± 62.20	158.0 ± 52.23	0.6839

HBsAg, hepatitis B surface antigen; AST, aspartate aminotransferase; ALT, alanine aminotransferase; WBC, white blood cells; PLT, platelet. The results are presented as the median inter-quartile range or mean ± standard deviation. Untreated CHB vs. CHB treated with Peg-IFN-α. UD, undetected; Bold values are statistically significant P < 0.05.

### Downregulation of ENPP1 and IFIT2 mRNA expression in untreated CHB patients

**Figure 1:**
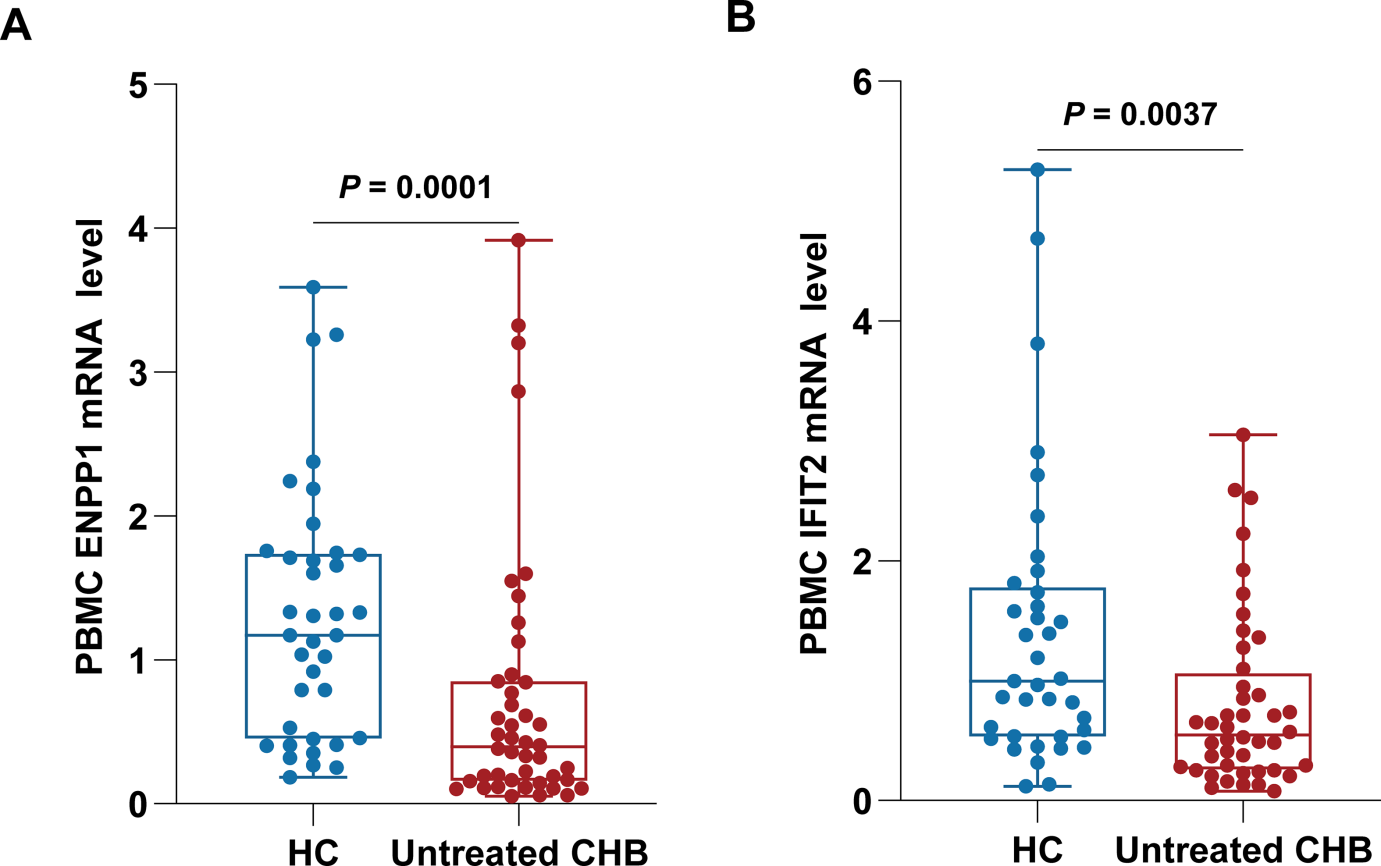
Baseline expression levels of ENPP1 and IFIT2 in PBMCs from untreated CHB patients versus healthy controls. **(A)** ENPP1 and **(B)** IFIT2 mRNA levels were quantified via qRT-PCR. Relative expression was determined using the 2^-ΔΔCt method normalized to GAPDH. Statistical significance was defined as P < 0.05.

To determine whether chronic HBV infection influences host gene expression, we evaluated mRNA levels of ENPP1 and IFIT2 in PBMCs by comparing untreated CHB patients (n = 44) with healthy individuals (n = 37). As illustrated in **Figure 1**, untreated CHB patients showed markedly lower transcript levels of ENPP1 (P = 0.0001; **Figure 1A**) and IFIT2 (P = 0.0037; **Figure 1B**) than healthy controls. These findings suggest that chronic HBV infection is associated with reduced expression of these ISGs at baseline.

### Comparison of clinical characteristics between responders and non-responders

**Figure 2:**
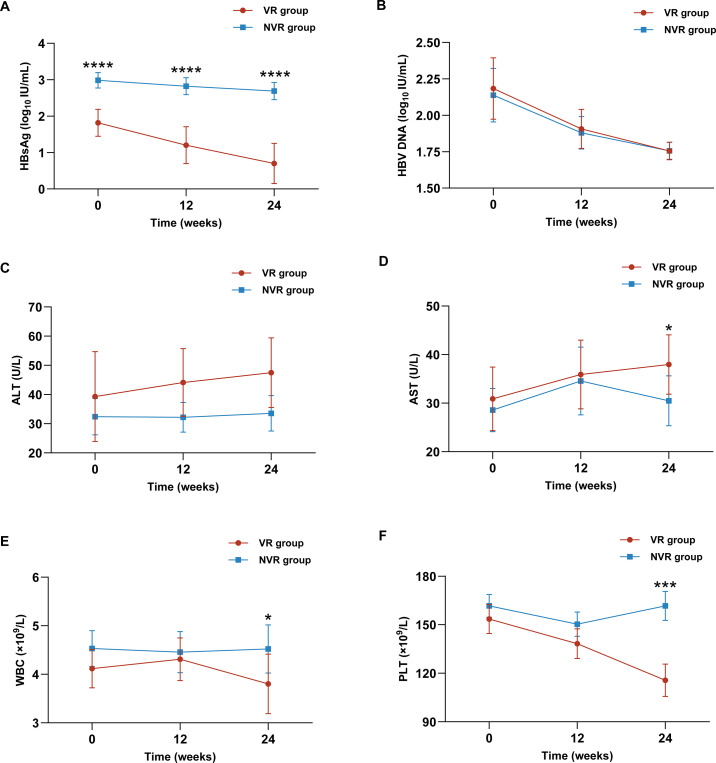
Longitudinal changes in clinical parameters in patients with and without virological response. Temporal changes in **(A)** HBsAg, **(B)** HBV DNA, **(C)** ALT, **(D)** AST, **(E)** WBC, and **(F)** PLT during the 48-week Peg-IFN-α treatment course; ●, indicates the median value for the VR group; ■, indicates median values for the NVR group; Error bars represent the interquartile range (25th–75th percentiles); *P < 0.05, **P < 0.01, ***P < 0.001, ****P < 0.0001.

As detailed in **Supplementary Table 6**, baseline demographic parameters (age, gender) and HBV genotype distribution were comparable between the VR and NVR groups (all P > 0.05). Patients who achieved a VR had notably lower serum HBsAg concentrations at weeks 0 (baseline), 12, and 24 compared with the NVR group (all P < 0.0001; **Figure 2**; **Supplementary Table 6**). No significant differences were observed in HBV DNA or ALT levels between the VR and NVR groups at any of these time points. AST, WBC, and PLT levels at baseline and week 12 were similar between the two groups; however, by week 24, the VR group exhibited reduced PLT and WBC counts (P = 0.0010 and P = 0.0117, respectively) alongside increased AST levels (P = 0.0121; **Figure 2**; **Supplementary Table 6**).

**Figure 3:**
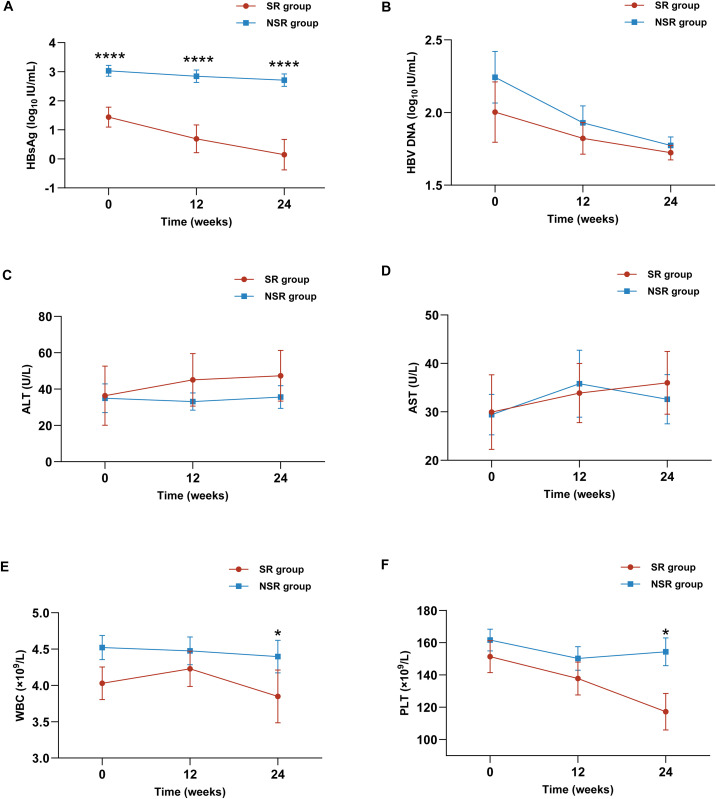
Longitudinal changes in clinical parameters in patients with and without serological response. Temporal changes in **(A)** HBsAg, **(B)** HBV DNA, **(C)** ALT, **(D)** AST, **(E)** WBC, and **(F)** PLT during the 48-week Peg-IFN-α treatment course; ●, indicates the median value for the SR group; ■, indicates median values for the NSR group; Error bars represent the interquartile range (25th–75th percentiles); *P < 0.05, **P < 0.01, ***P < 0.001, ****P < 0.0001.

Similarly, baseline characteristics including age, gender, and HBV genotype were comparable between the SR and NSR groups (all P > 0.05; **Supplementary Table 7**). Serum HBsAg levels were consistently lower in the SR group than in the NSR group at weeks 0, 12, and 24, with all comparisons reaching strong statistical significance (all P < 0.0001; **Figure 3**; **Supplementary Table 7**). No significant between-group differences were detected for HBV DNA, ALT, or AST at any time point during therapy. PLT and WBC counts were comparable between the two groups at baseline and week 12; however, both parameters declined to significantly lower levels in the SR group by week 24 (P = 0.0183 and P = 0.0373, respectively; **Figure 3**; **Supplementary Table 7**).

### Dynamic changes of ENPP1 and IFIT2 mRNA levels in PBMCs of CHB patients receiving Peg-IFN-α during the early antiviral therapy

**Figure 4:**
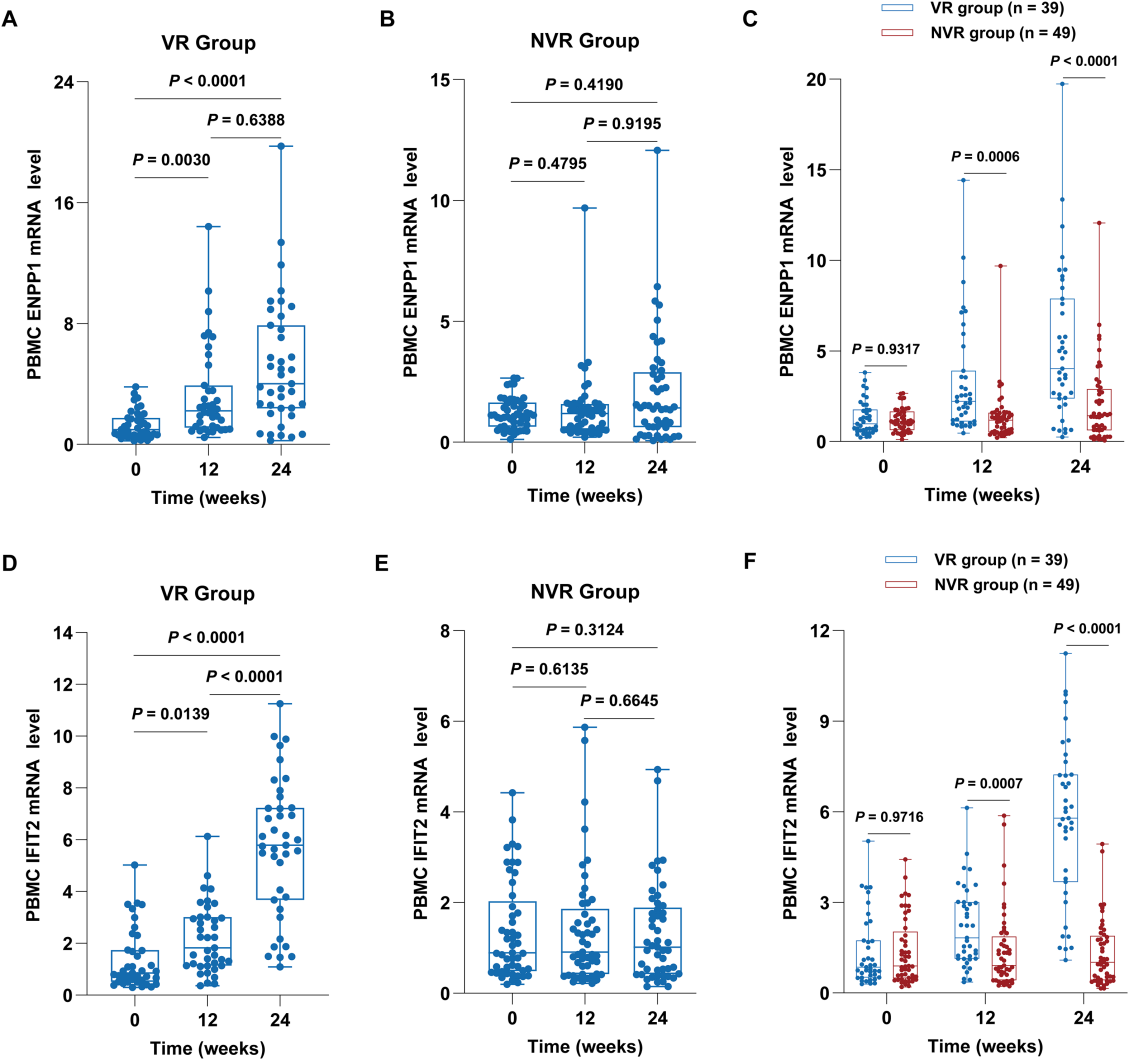
On-treatment dynamics of ENPP1 and IFIT2 mRNA expression in VR and NVR groups. Time changes in ENPP1 mRNA levels during therapy are shown for the VR group **(A)** and the NVR group **(B)**, while corresponding changes in IFIT2 mRNA levels are presented for the VR group **(D)** and the NVR group **(E)**. Comparative analyses of ENPP1 **(C)** and IFIT2 **(F)** mRNA expression between VR and NVR groups were performed at baseline, week 12, and week 24 of treatment. Data are presented as median (interquartile range). Statistical significance was defined as P < 0.05.

To investigate the dynamics of ENPP1 and IFIT2 expression in PBMCs during the early phase of Peg-IFN-α therapy, samples were obtained from patients in the VR and NVR groups at weeks 0, 12, and 24. Across the treatment period, both ENPP1 and IFIT2 mRNA levels generally increased; however, the response kinetics diverged notably between responders and non-responders. Among patients in the VR group, ENPP1 mRNA levels exhibited a significant surge from baseline to week 12 (P = 0.0030) and continued to increase through week 24 (P < 0.0001; **Figure 4A**). A similar trend was noted for IFIT2, which showed significant elevation in the VR group at both week 12 (P = 0.0139) and week 24 (P < 0.0001; **Figure 4D**). Conversely, patients in the NVR group failed to show statistically significant fluctuations in either ENPP1 or IFIT2 mRNA expression across the measured time points (all P > 0.05; **Figures 4B** and **4E**). To further assess group differences, mRNA expression of ENPP1 and IFIT2 was analyzed in PBMCs from VR and NVR patients. ENPP1 transcript levels were higher in the VR group at week 12 (P = 0.0006) and further increased at week 24 (P < 0.0001; **Figure 4C**). Similarly, IFIT2 mRNA levels were significantly elevated in the VR group compared with the NVR group at both week 12 (P = 0.0007) and week 24 (P < 0.0001; **Figure 4F**).

**Figure 5:**
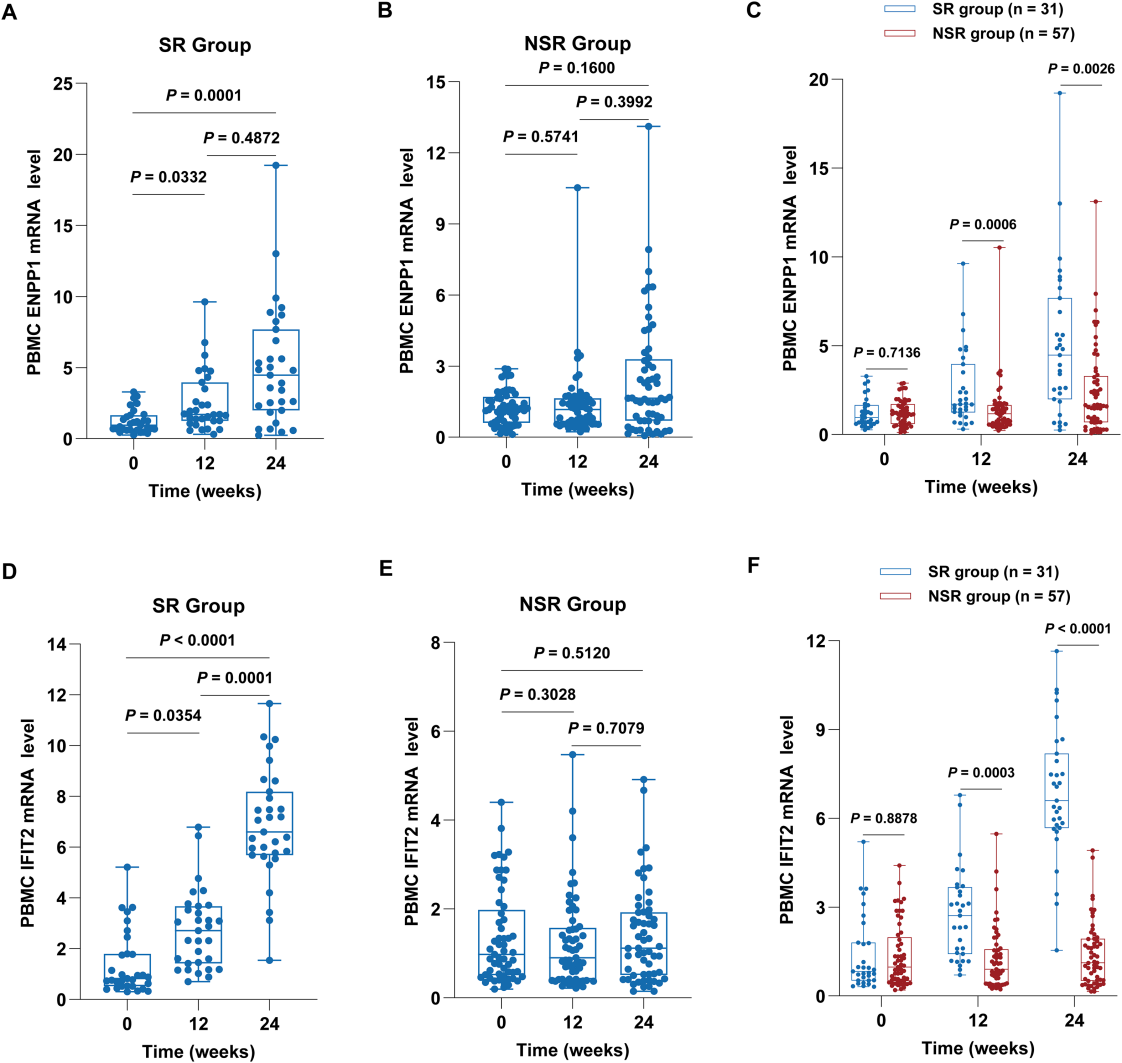
On-treatment dynamics of ENPP1 and IFIT2 mRNA expression in SR and NSR groups. Time changes in ENPP1 mRNA levels during therapy are shown for the SR group **(A)** and the NSR group **(B)**, while corresponding changes in IFIT2 mRNA levels are presented for the SR group **(D)** and the NSR group **(E)**. Comparative analyses of ENPP1 **(C)** and IFIT2 **(F)** mRNA expression between SR and NSR groups were performed at baseline, week 12, and week 24 of treatment. Data are presented as median (interquartile range). Statistical significance was defined as P < 0.05.

We subsequently assessed whether these kinetic patterns aligned with serological outcomes. In the SR group, ENPP1 mRNA was significantly upregulated at week 12 (P = 0.0332) and week 24 (P = 0.0001; **Figure 5A**) relative to baseline. IFIT2 followed a similar pattern, with marked increases observed at week 12 (P = 0.0354) and week 24 (P < 0.0001; **Figure 5D**). In contrast, the NSR group showed no significant changes in ENPP1 or IFIT2 expression throughout treatment (all P > 0.05; **Figures 5B, E**). When comparing the two groups, ENPP1 mRNA levels were significantly higher in SR patients than in NSR patients at week 12 (P = 0.0006) and week 24 (P = 0.0026; **Figure 5C**). A similar pattern was observed for IFIT2, with the SR group showing significantly elevated levels relative to the NSR group at week 12 (P = 0.0003) and week 24 (P < 0.0001; **Figure 5F**). To further distinguish between HBsAg loss and complete serological conversion, the 31 patients who achieved serological response (SR) were subdivided into those with isolated HBsAg loss (n = 20) and those with anti-HBs seroconversion (n = 11). At baseline (week 0), no significant differences in ENPP1 or IFIT2 mRNA expression were observed among the NSR (n = 57), isolated HBsAg loss, and anti-HBs seroconversion groups. By week 12, both responder subgroups exhibited significantly increased ENPP1 and IFIT2 expression relative to the NSR group. At week 24, patients who achieved anti-HBs seroconversion exhibited significantly higher ENPP1 (P = 0.0012) and IFIT2 (P = 0.0097) mRNA levels than those with isolated HBsAg loss (**Supplementary Figure 3**). These findings suggest that more sustained upregulation of ENPP1 and IFIT2 during Peg-IFN-α therapy may be associated with a more complete serological response. To evaluate whether these dynamic upregulation patterns were modified by viral genotype, we conducted exploratory stratified analyses according to HBV genotype (Genotype B vs. Genotype C). As shown in **Supplementary Figures 4** and **S5**, within both genotype B and genotype C subgroups, patients who achieved VR or SR exhibited consistently higher ENPP1 and IFIT2 mRNA levels at weeks 12 and 24 compared with non-responders. Notably, this upregulation pattern was consistently observed across both genotypes and clinical endpoints. These stratified findings confirmed that the early induction of ENPP1 and IFIT2 reflects a fundamental host immune response, suggesting that their predictive performance is largely independent of HBV genotype.

### Correlation between ENPP1 and IFIT2 mRNA levels and HBsAg reduction during Peg-IFN-α therapy

**Figure 6:**
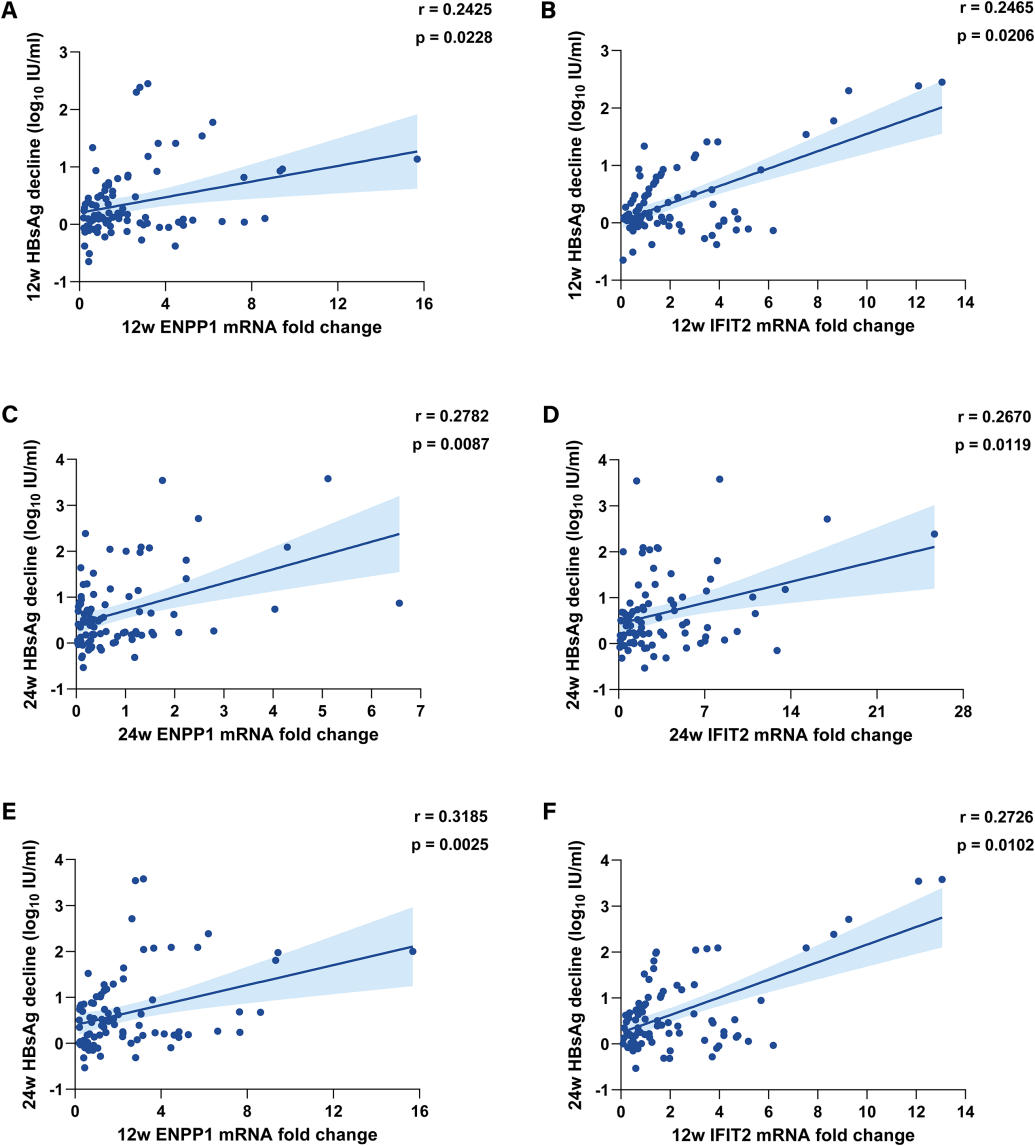
Correlation between ENPP1 and IFIT2 mRNA levels and HBsAg reduction during Peg-IFN-α therapy. **(A, C)** Correlations between ENPP1 mRNA levels and HBsAg reduction at week 12 and week 24. **(B, D)** Correlations between IFIT2 mRNA levels and HBsAg reduction at week 12 and week 24. **(E, F)** Correlations between ENPP1 and IFIT2 mRNA levels at week 12 and HBsAg reduction at week 24. ENPP1 and IFIT2 mRNA levels in PBMCs were measured by qRT-PCR and calculated using the 2^-ΔΔCt method (Livak method), with GAPDH as the reference gene. HBsAg levels were log10 transformed. Correlation analyses were performed using Spearman’s rank correlation analysis. The correlation coefficient (r) and two-tailed P values were calculated via Spearman’s rank correlation. P < 0.05 was considered statistically significant.

We evaluated the association between the mRNA expression of ENPP1 and IFIT2 and HBsAg reduction during therapy. At week 12, both ENPP1 (r = 0.2425, P = 0.0228, **Figure 6A**) and IFIT2 (r = 0.2465, P = 0.0206, **Figure 6B**) levels exhibited a significant positive correlation with concurrent HBsAg reduction. Furthermore, this positive correlation persisted at week 24 for both ENPP1 (r = 0.2782, P = 0.0087, **Figure 6C**) and IFIT2 (r = 0.2670, P = 0.0119, **Figure 6D**). To further investigate the temporal relationship between early gene expression and subsequent HBsAg changes, time-lagged correlation analyses were performed. Notably, the early expression of ENPP1 (r = 0.3185, P = 0.0025, **Figure 6E**) and IFIT2 (r = 0.2726, P = 0.0102, **Figure 6F**) at week 12 was significantly correlated with the HBsAg reduction at week 24.

### Association of ENPP1 and IFIT2 with virological and serological responses to Peg-IFN-α therapy

To determine factors associated with therapeutic efficacy, we evaluated the relationship between ENPP1, IFIT2, and clinical responses using univariate and multivariate logistic regression. As detailed in **Supplementary Table 8**, univariate analyses showed that baseline HBsAg, along with HBsAg, ENPP1, and IFIT2 levels at weeks 12 and 24, correlated significantly with VR. Additionally, ALT and PLT levels at week 24 were identified as significant factors. Multivariate logistic regression included clinically relevant covariates together with variables selected from the univariate analysis (P < 0.10). As presented in **Supplementary Table 8**, multivariate analyses further confirmed that baseline HBsAg level remained independently associated with VR at week 0 (adjusted odds ratio [aOR] = 0.116, P < 0.0001). At week 12, HBsAg (aOR = 0.341, P = 0.0001), ENPP1 (aOR = 1.805, P = 0.0092), and IFIT2 (aOR = 1.766, P = 0.0119) were independently associated with VR. Moreover, HBsAg (aOR = 0.445, P = 0.0137), ENPP1 (aOR = 1.560, P = 0.0026), and IFIT2 (aOR = 2.394, P = 0.0011) measured at week 24 maintained independent significance.

Similar patterns were observed for SR. As detailed in **Supplementary Table 9**, univariate analyses revealed a significant association between baseline HBsAg levels and SR at week 48. Furthermore, HBsAg, ENPP1, and IFIT2 levels at weeks 12 and 24 were also correlated with SR, while PLT levels at week 24 showed an additional association with SR. Multivariate analyses presented in **Supplementary Table 9**, after adjusting for corresponding clinically relevant variables and those with P < 0.10 in univariate analyses, indicated that HBsAg was independently associated with SR (aOR = 0.075, P < 0.0001) at baseline. Independent predictors of SR at week 12 included HBsAg (aOR = 0.074, P < 0.0001), ENPP1 (aOR = 1.739, P = 0.0232), and IFIT2 (aOR = 2.642, P = 0.0033). Additionally, HBsAg (aOR = 0.145, P < 0.0001), ENPP1 (aOR = 1.428, P = 0.0109), and IFIT2 (aOR = 4.299, P = 0.0056) measured at week 24 were also identified as independent factors associated with SR.

### Predictive performance of ENPP1 and IFIT2 mRNA levels for virological and serological responses

Receiver operating characteristic (ROC) analysis was performed to assess the ability of ENPP1 and IFIT2 mRNA levels at weeks 0, 12, and 24 to predict virological and serological responses following 48 weeks of Peg-IFN-α therapy.

**Figure 7:**
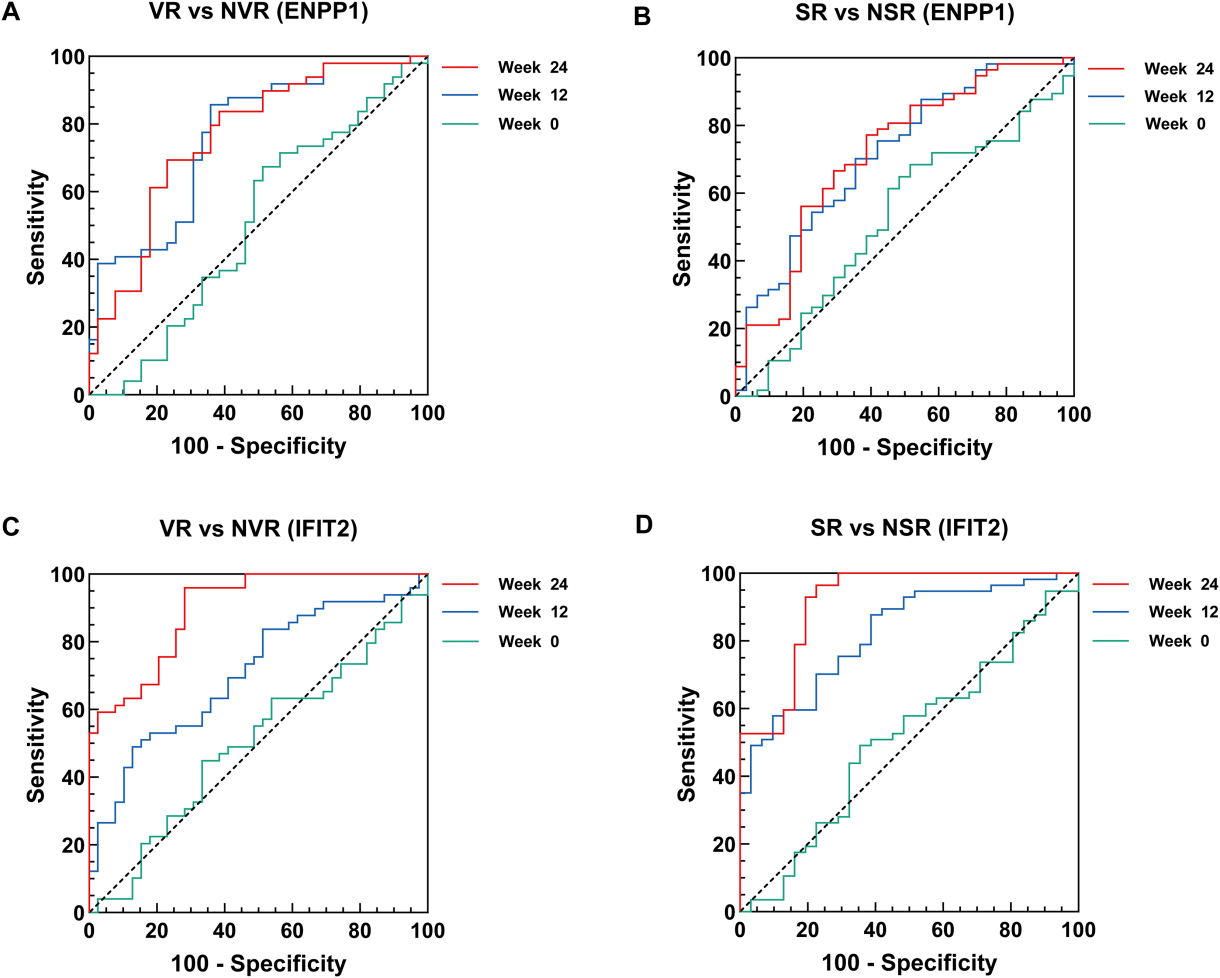
Assessment of predictive performance using Receiver Operating Characteristic (ROC) analysis. **(A, B)** ROC curves illustrating the accuracy of ENPP1 at weeks 0, 12, and 24 for predicting virological and serological response. **(C, D)** ROC curves for IFIT2 at weeks 0, 12, and 24. Optimal cut-offs were determined using the Youden index. AUC, Area Under the Curve.

For VR prediction, ENPP1 mRNA levels at week 12 showed the highest predictive accuracy among the tested time points, with an area under the curve (AUC) of 0.7645 (P < 0.0001; **Figure 7A**; **Supplementary Table 10**). Based on the Youden index, a cut-off of 1.7789 provided a sensitivity of 64.10% and specificity of 85.70%. Conversely, for SR, the best predictive performance was observed at week 24 (AUC = 0.7399, P = 0.0007). Here, a cut-off of 3.3767 corresponded to 61.40% sensitivity and 77.20% specificity (**Figure 7B**, **Supplementary Table 10**). For IFIT2, the week 24 measurement offered superior prognostic value for VR, achieving an AUC of 0.8791 (P < 0.0001). An optimal cut-off of 2.9699 resulted in 82.10% sensitivity and 95.90% specificity (**Figure 7C**; **Supplementary Table 10**). Similarly, week 24 IFIT2 levels were comparably effective in predicting SR (AUC = 0.8879, P < 0.0001); a cut-off of 3.4042 achieved 93.50% sensitivity and 96.50% specificity (**Figure 7D**; **Supplementary Table 10**).

### Predictive performance of combined ENPP1 and IFIT2 models for treatment response

Based on the ROC analyses of individual ENPP1 and IFIT2 mRNA levels at different treatment time points, combined prediction models were established using the optimal time points that demonstrated the highest discriminative performance for each response endpoint.

**Figure 8:**
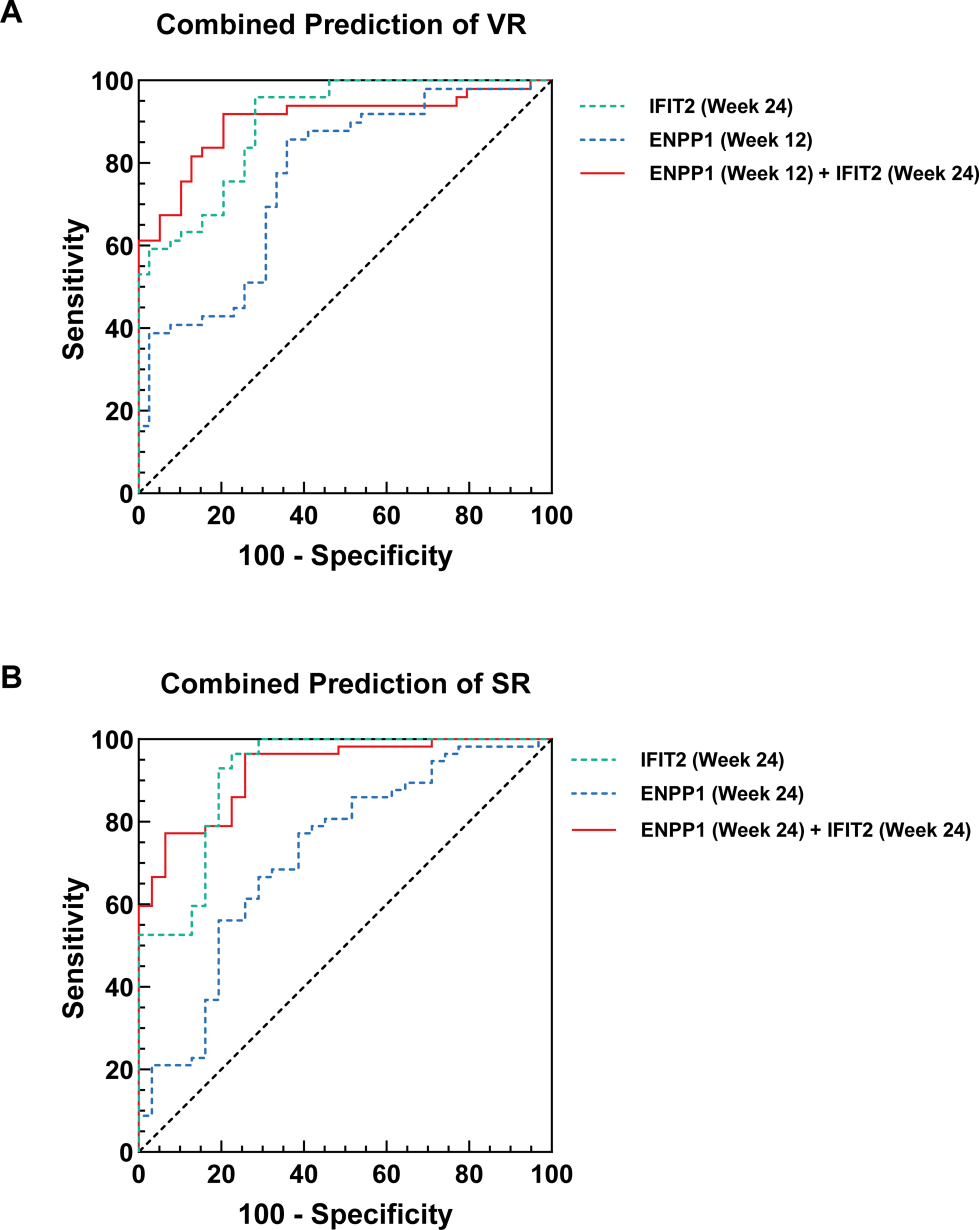
ROC curves for combinatorial prediction models. **(A)** Predictive accuracy of the sequential model combining ENPP1 (week 12) and IFIT2 (week 24) for virological response. **(B)** Performance of the dual-marker model combining ENPP1 (week 24) and IFIT2 (week 24) for predicting serological response.

For virological response, ROC analyses revealed distinct optimal time points for the two genes, with ENPP1 showing the strongest predictive performance at week 12, while IFIT2 demonstrated superior discrimination at week 24. Accordingly, a sequential prediction model combining ENPP1 at week 12 and IFIT2 at week 24 was constructed. This model achieved the highest predictive accuracy, yielding an AUC of 0.9098 (P < 0.0001), a sensitivity of 92.30%, and a specificity of 83.70% (**Figure 8A**; **Supplementary Table 11**), significantly exceeding the performance of either ENPP1 alone (AUC = 0.7645) or IFIT2 alone (AUC = 0.8791) for VR prediction.

For serological response, both ENPP1 and IFIT2 exhibited their highest predictive accuracy at week 24. Accordingly, a dual-marker model integrating ENPP1 and IFIT2 mRNA levels measured at week 24 was established. This model achieved an AUC of 0.9217 (P < 0.0001), with a sensitivity of 87.10% and a specificity of 98.20%, significantly outperforming either ENPP1 alone (AUC = 0.7399) or IFIT2 alone (AUC = 0.8879) (**Figure 8B**; **Supplementary Table 11**).

### Validation of the combined predictive models in external and internal cohorts

**Figure 9:**
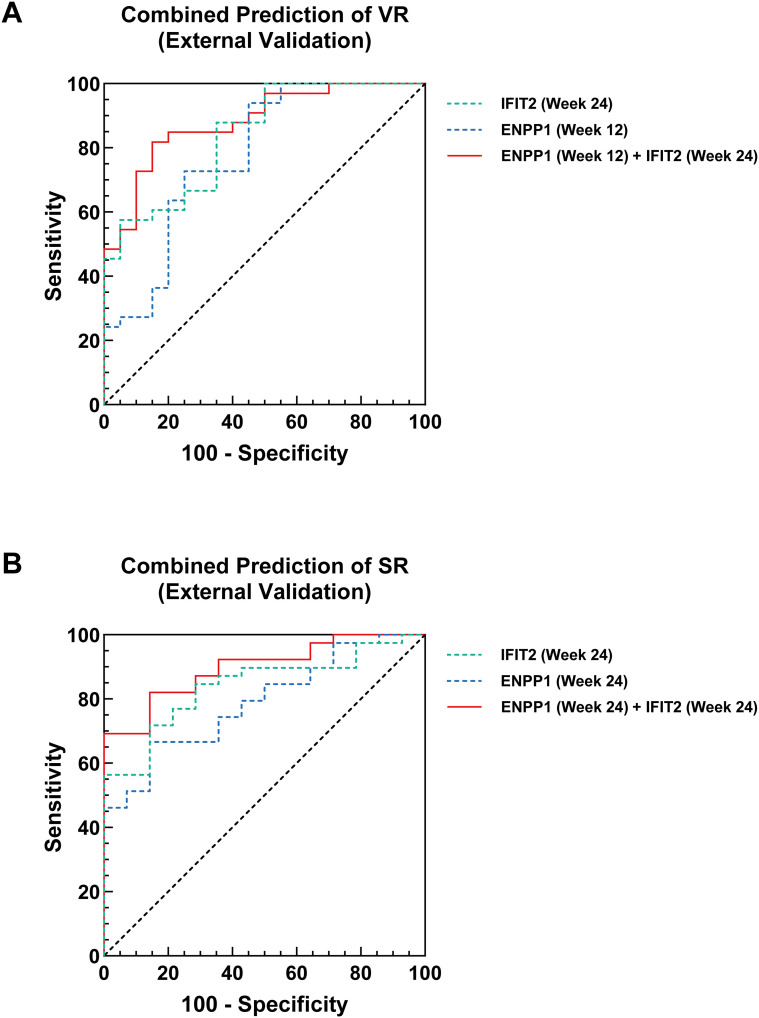
Predictive performance of the combined models in the independent external validation cohort. **(A)** ROC curve of the sequential model (integrating ENPP1 at week 12 and IFIT2 at week 24) for predicting virological response. **(B)** ROC curve of the dual-marker model (integrating ENPP1 and IFIT2 at week 24) for predicting serological response.

**Figure 10:**
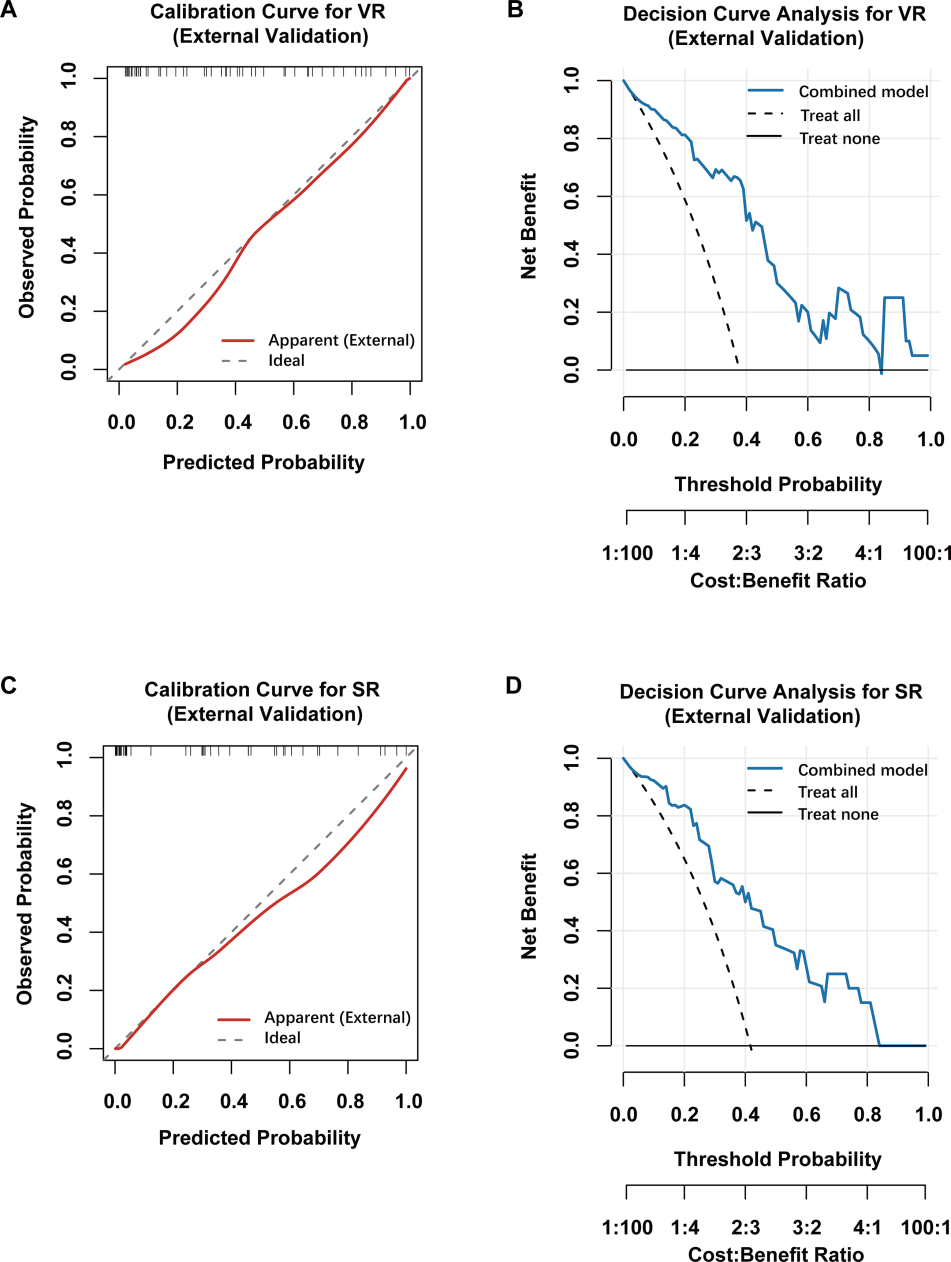
Calibration and clinical utility of the combined prediction models in the external validation cohort. **(A)** Calibration curve and **(B)** Decision curve analysis (DCA) for the sequential model in predicting virological response. **(C)** Calibration curve and **(D)** DCA for the dual-marker model in predicting serological response.

To further evaluate the generalizability of the predictive models, an independent external validation cohort, completely separate from and not involved in model development, was analyzed. For VR prediction, the combined model integrating ENPP1 at week 12 and IFIT2 at week 24 demonstrated robust discriminative performance, with an AUC of 0.8818 (95% CI: 0.7918-0.9718, P < 0.0001), a sensitivity of 81.30%, and a specificity of 84.85% at the prespecified cut-off (**Figure 9A**; **Supplementary Table 12**). Similarly, for SR prediction, the combined model based on ENPP1 and IFIT2 at week 24 yielded an AUC of 0.8974 (95% CI: 0.8146-0.9803, P < 0.0001), with a sensitivity of 78.57% and a specificity of 82.05% using the predefined cut-off (**Figure 9B**; **Supplementary Table 12**). Notably, all cut-off values were directly derived from the primary training cohort and applied without re-estimation in the external validation cohort. Calibration analysis in the external validation cohort demonstrated good agreement between predicted and observed outcomes. For VR prediction, the calibration intercept and slope were 0.016 and 1.091, respectively, with a Brier score of 0.1386; for SR prediction, the corresponding values were 0.031 and 1.028, with a Brier score of 0.1161 (**Supplementary Table 13**). Visual inspection of the calibration curves further supported these findings, showing close agreement between predicted and observed probabilities (**Figures 10A, C**). Decision curve analysis (DCA) demonstrated that the combined models provided a favorable net clinical benefit across a range of threshold probabilities for both VR and SR (**Figures 10B, D**).

Internal validation using bootstrap resampling confirmed the robustness of the predictive models. The optimism-corrected C-index was 0.8823 for VR prediction and 0.9054 for SR prediction. Calibration performance remained stable, with slopes of 0.9983 and 1.1083, intercepts of 0.0026 and 0.0031, and Brier scores of 0.1125 and 0.1078 for VR and SR, respectively. The calibration curves further demonstrated good agreement between predicted and observed outcomes, and DCA indicated consistent net clinical benefit (**Supplementary Figure 6**; **Supplementary Table 14**). Overall, these results indicate that the combined models maintain stable discriminative ability, calibration performance, and potential clinical utility across both internal and external validation settings, supporting their robustness and external applicability.

### Predictive performance of ENPP1 and IFIT2 compared with baseline HBsAg and HBsAg decline at week 12

To determine whether ENPP1 and IFIT2 provide predictive value beyond conventional markers, their individual discriminative performance was compared with baseline HBsAg and HBsAg decline at week 12. For VR prediction, ENPP1 at week 12 (AUC = 0.7645) and IFIT2 at week 12 (AUC = 0.7080) showed predictive performance comparable to baseline HBsAg alone (AUC = 0.7393), with no statistically significant differences (P = 0.6531 and P = 0.8492, respectively). Similarly, ENPP1 at week 24 (AUC = 0.7298) also demonstrated no significant difference compared with baseline HBsAg (P = 0.5359). In contrast, IFIT2 at week 24 exhibited significantly superior predictive capability for VR, achieving an AUC of 0.8791 compared with 0.7393 for baseline HBsAg (P = 0.0103; **Supplementary Figure 7** and **Supplementary Table 15**). For SR prediction, ENPP1 at weeks 12 and 24 yielded AUCs of 0.7102 and 0.7399, respectively, both comparable to baseline HBsAg (AUC = 0.7266), with no statistically significant differences (P = 0.7652 and P = 0.9634, respectively). By contrast, IFIT2 demonstrated significantly improved predictive performance for SR at both week 12 (AUC = 0.8223; P = 0.0138) and week 24 (AUC = 0.8879; P = 0.0078) compared with baseline HBsAg (**Supplementary Figure 7** and **Supplementary Table 15**).

We further compared the predictive performance of ENPP1 and IFIT2 with HBsAg decline at week 12. For VR prediction, ENPP1 at week 12 (AUC = 0.7645) showed comparable predictive performance to HBsAg decline at week 12 (AUC = 0.7758; P = 0.7592), whereas IFIT2 at week 12 exhibited significantly lower predictive accuracy (AUC = 0.7080; P = 0.0453). ENPP1 at week 24 remained comparable to HBsAg decline at week 12 (P = 0.4539), while IFIT2 at week 24 demonstrated significantly improved discrimination with an AUC of 0.8791 (P = 0.0325; **Supplementary Figure 8** and **Supplementary Table 16**). For SR prediction, ENPP1 at week 12 and week 24 demonstrated significantly lower predictive performance than HBsAg decline at week 12 (P = 0.0268 and P = 0.0486, respectively). However, IFIT2 at week 12 achieved an AUC of 0.8223, comparable to HBsAg decline at week 12 (AUC = 0.8270; P = 0.2960). Notably, IFIT2 at week 24 yielded the highest predictive accuracy for SR (AUC = 0.8879), significantly outperforming HBsAg decline at week 12 (P = 0.0375; **Supplementary Figure 8** and **Supplementary Table 16**).

### Incremental value of ENPP1 and IFIT2 in predicting treatment response

To further assess the incremental predictive value of combining ENPP1 and IFIT2 with conventional HBsAg parameters, integrated models were constructed and their performance was evaluated. For VR prediction, the sequential model integrating ENPP1 at week 12 and IFIT2 at week 24 achieved an AUC of 0.9098, significantly higher than baseline HBsAg alone (AUC = 0.7393; P = 0.0076). After incorporation of baseline HBsAg into the combined model, the predictive performance further increased to an AUC of 0.9391, representing significantly improved discriminative performance compared with baseline HBsAg alone (P = 0.0095), with a sensitivity of 84.60% and a specificity of 91.80% (**Supplementary Figure 9** and **Supplementary Table 17**). In addition, compared with baseline HBsAg alone, the integrated model demonstrated significant incremental discrimination, with a continuous net reclassification improvement (NRI) of 0.948 (95% CI: 0.563-1.333, P < 0.0001) and an integrated discrimination improvement (IDI) of 0.241 (95% CI: 0.156-0.326, P < 0.0001, **Supplementary Table 18**). For SR prediction, the combined model integrating ENPP1 and IFIT2 at week 24 achieved an AUC of 0.9217, significantly exceeding that of baseline HBsAg alone (AUC = 0.7266; P = 0.0155). After incorporation of baseline HBsAg into the combined model, the predictive performance further increased to an AUC of 0.9343, representing significantly improved discriminative performance compared with baseline HBsAg alone (P = 0.0076), with a sensitivity of 87.10% and a specificity of 86.00% (**Supplementary Figure 9** and **Supplementary Table 17**). Furthermore, compared with baseline HBsAg alone, the integrated model demonstrated substantial reclassification improvement, with a continuous NRI of 1.072 (95% CI: 0.648-1.496, P = 0.0019) and an IDI of 0.279 (95% CI: 0.184-0.374, P = 0.0012, **Supplementary Table 18**).

When HBsAg decline at week 12 was used for VR prediction, the combined model incorporating ENPP1 and IFIT2 demonstrated significantly improved predictive performance, with the AUC increasing from 0.7758 to 0.9098 (P = 0.0205). The addition of HBsAg decline at week 12 to the dual-gene model further improved the AUC to 0.9214 (P = 0.0149), with a sensitivity of 92.30% and a specificity of 85.70% (**Supplementary Figure 10** and **Supplementary Table 19**). Incremental analysis demonstrated a continuous NRI of 0.603 (95% CI: 0.281-0.925, P = 0.0026) and an IDI of 0.138 (95% CI: 0.071-0.205, P = 0.0037, **Supplementary Table 20**), indicating additional predictive value beyond HBsAg decline at week 12 alone. Similarly, when compared with HBsAg decline at week 12 alone (AUC = 0.8270), the ENPP1 and IFIT2 combined model at week 24 demonstrated significantly improved predictive discrimination for SR (AUC = 0.9217; P = 0.0236). Incorporation of HBsAg decline at week 12 into the dual-gene model further improved the AUC to 0.9315 (P = 0.0152), yielding a sensitivity of 80.60% and a specificity of 91.60% (**Supplementary Figure 10** and **Supplementary Table 19**). Incremental analyses further confirmed the added predictive value of the integrated model, with a continuous NRI of 0.572 (95% CI: 0.231-0.913, P = 0.0045) and an IDI of 0.119 (95% CI: 0.057-0.181, P = 0.0061, **Supplementary Table 20**). These findings indicate that integrating ENPP1 and IFIT2 with HBsAg substantially enhances the prediction of both virological and serological responses during Peg-IFN-α therapy.

## Discussion

Pegylated interferon alpha (Peg-IFN-α) is the primary treatment for chronic hepatitis B (CHB) according to clinical guidelines, showing potential for better outcomes and a clinical cure (7, 34). However, the substantial heterogeneity in clinical response underscores the critical need for reliable early biomarkers to predict treatment efficacy, optimize patient stratification, and reduce unnecessary adverse effects and healthcare costs. In this study, we investigated the dynamic changes of ENPP1 and IFIT2 mRNA in PBMCs during Peg-IFN-α therapy in HBeAg-negative CHB patients. Our findings demonstrate that both genes are dynamically regulated during treatment and are closely associated with virological and serological outcomes, suggesting their utility as valuable host immune markers for monitoring antiviral efficacy. Despite previous studies characterizing ENPP1 and IFIT2 as ISGs involved in antiviral immune responses (27, 29), the present study evaluated their dynamic expression profiles and clinical predictive value in HBeAg-negative CHB patients receiving Peg-IFN-α therapy. By extending these mechanistic insights into a prospective, longitudinally monitored clinical setting, this study provides a translational perspective linking ISG activity with treatment response prediction.

Our first major finding was the significant suppression of ENPP1 and IFIT2 in untreated CHB patients compared to healthy controls. This observation is consistent with the concept of viral-induced innate immune dysfunction in chronic HBV infection. Recent studies have similarly reported that Toll-like receptor 8 (TLR8) (35) and TRIM family proteins (TRIM19/38) (20, 36) are compromised in CHB patients. We speculate that this downregulation reflects viral-mediated immune modulation, potentially through interference with upstream signaling pathways such as JAK–STAT, leading to dampened expression of antiviral restriction factors. Consequently, the impaired baseline expression of ENPP1 and IFIT2 indicates a state of immune exhaustion, suggesting that Peg-IFN-α therapy may act to reverse this suppression and reinvigorate the antiviral immune state.

Consistent with this hypothesis, dynamic analyses revealed that ENPP1 and IFIT2 mRNA levels increased selectively in patients who achieved virological response (VR) or serological response (SR), with significant divergence between responders and non-responders evident as early as week 12 and maintained at week 24. This response-specific induction pattern suggests that Peg-IFN-α–mediated upregulation of these genes represents an effective functional restoration of host immunity, rather than nonspecific interferon exposure. The absence of similar changes in NVR and NSR patients further supports their utility as discriminative biomarkers. Regarding the overall treatment efficacy, we observed a virological response in 39 of 88 patients (44.32%) and a serological response in 31 patients (35.23%) after 48 weeks of Peg-IFN-α therapy. These rates are comparable to, or slightly better than, historical cohorts. For instance, Huang et al. reported HBsAg seroconversion rates of 27.9% (37), and studies on NUCs-experienced patients switching to Peg-IFN reported rates of 22.2–26.5% (14, 38). The slightly higher rates in our study may reflect the inclusion of patients with evidence of subtle immune activity and the use of response criteria tailored to this low-viremia population. The consistency of our response rates with published data supports the representativeness of our cohort and strengthens the clinical relevance of our subsequent biomarker analyses. Although Peg-IFN-α therapy is associated with potential adverse effects and is not routinely recommended by current AASLD/EASL guidelines for all HBeAg-negative patients with low-level viremia and normal alanine aminotransferase levels, treatment in this cohort was implemented as an individualized, functional cure-oriented strategy in selected patients under careful clinical evaluation and informed consent. Most patients in this cohort had additional clinical features supporting proactive intervention, including older age, family history of HBV-related cirrhosis or HCC, and mild fibrosis. This approach aligns with recent Chinese expert consensuses emphasizing early intervention in selected advantageous populations to maximize the probability of clinical cure (7, 39).

We also monitored the dynamic changes in serum HBsAg, HBV DNA, ALT, AST, WBC, and PLT at various time points following treatment. Compared to the NSR and NVR groups, serum HBsAg levels in the SR and VR groups were significantly reduced after treatment initiation, supporting the clinical relevance of virological and serological endpoints used in this study. Despite being a preferred treatment approach, Peg-IFN-α therapy shows unsatisfactory HBsAg seroclearance rates among HBeAg-negative CHB patients. Thus, investigating early and reliable predictors of Peg-IFN-α efficacy remains of great clinical significance. Our correlation analyses provide further insight into the temporal dynamics between ISG expression and viral antigen decline during Peg-IFN-α therapy. The significant positive correlations observed between ENPP1 and IFIT2 mRNA levels and HBsAg reduction at both weeks 12 and 24 suggest that sustained ISG activation is integral to ongoing antigen clearance. More importantly, time-lagged analyses demonstrated that ENPP1 and IFIT2 expression at week 12 was significantly associated with subsequent HBsAg reduction at week 24. This finding implies that robust early immune activation serves as a prerequisite for subsequent virological and serological responses, highlighting the potential of ENPP1 and IFIT2 as early on-treatment indicators of therapeutic efficacy. Although the correlations between individual ISG levels and HBsAg decline were moderate, week 12 represents a clinically established milestone for response-guided Peg-IFN-α strategies (40, 41). Importantly, the early divergence in ENPP1 and IFIT2 expression between responders and non-responders observed at week 12 suggests that these ISGs may provide additional information regarding host immune activation during therapy. The inclusion of earlier sampling time points, such as week 4, may further improve the temporal resolution and clinical utility of ISG-based prediction during the initial phase of treatment.

Previous clinical studies have identified various factors associated with VR or SR in CHB patients undergoing Peg-IFN-α therapy, including gender, age, ALT, AST, HBsAg, HBeAg, and HBV DNA (42). Our current study, through both univariate and multivariate analyses, identified ENPP1 and IFIT2 mRNA levels as independent factors associated with SR and VR. Receiver operating characteristic (ROC) analyses further demonstrated that time point–specific measurements of these markers during therapy provided meaningful discriminative performance, reinforcing their potential role as early predictive biomarkers of Peg-IFN-α response. While the identification of individual biomarkers is valuable, the complexity of the host immune response against HBV suggests that single-marker approaches may lack the sensitivity and specificity required for precision medicine. Therefore, we developed combinatorial models to maximize predictive accuracy. For virological response, our sequential prediction model integrating ENPP1 at week 12 and IFIT2 at week 24 achieved an AUC of 0.9098. The improved performance of this model suggests that coordinated induction of multiple ISGs at distinct treatment phases may provide a more robust indicator of viral suppression. For serological response, our dual-marker model combining ENPP1 and IFIT2 measured at week 24 achieved a high AUC of 0.9217. Given that serological response reflects sustained immune-mediated viral control rather than rapid suppression of viral replication, this combined model may capture complementary aspects of immune activation during Peg-IFN-α therapy, thereby improving discrimination compared with individual markers alone. The superior performance of these combined models over individual markers underscores the value of multi-gene assessment in capturing the multifaceted immune response to Peg-IFN-α. Previous mechanistic studies have shown that ENPP1 suppresses HBV EnhII/BCP promoter activity and inhibits pregenomic RNA transcription, thereby restricting early viral replication (27). In contrast, IFIT2 has been reported to target the SP1 and SP2 promoters of HBV, resulting in reduced transcription of surface antigen mRNAs (29). These findings suggest that ENPP1 and IFIT2 may represent distinct but complementary antiviral pathways during Peg-IFN-α therapy. Interestingly, our temporal analyses appeared to further support this biological distinction. ENPP1 expression at week 12 demonstrated the strongest predictive value for virological response, which is consistent with its proposed role in the early suppression of viral replication. In contrast, IFIT2 expression at week 24 showed superior predictive performance for both virological and serological responses, potentially reflecting its association with sustained antigen reduction during later treatment stages. Therefore, the combined assessment of ENPP1 and IFIT2 may simultaneously capture different phases of interferon-mediated antiviral activity, which may partly explain the superior discriminative performance of the combined models compared with either marker alone. Importantly, the robustness and generalizability of the predictive models were supported by both external and internal validation analyses. In the independent external cohort, the combined models maintained high discriminative performance for both VR and SR, with AUC values approaching 0.90. Calibration analyses showed good agreement between predicted and observed outcomes, and decision curve analysis confirmed a consistent net clinical benefit across a wide range of threshold probabilities. Together, these findings indicate that the models are not only statistically reliable but also retain their predictive utility beyond the original training cohort. Consistent with these observations, internal validation using bootstrap resampling further minimized the risk of overfitting. The high optimism-corrected C-index and stable calibration parameters suggest that the observed predictive performance is unlikely to be attributable to random variation within the training dataset. The close agreement between apparent and corrected estimates further supports the stability of the models. The ability of the combined models to maintain performance across different validation settings may be related to the biological involvement of these ISGs in antiviral immune responses. From a clinical perspective, early identification of patients with a high likelihood of response could facilitate individualized therapeutic decision-making, particularly in guiding treatment continuation or modification during Peg-IFN-α therapy.

To further operationalize this individualized approach, the predictive value of ENPP1 and IFIT2 should be interpreted within an on-treatment dynamic stratification framework. Peg-IFN-α therapy in chronic hepatitis B is largely guided by longitudinal virological and serological responses, yet early identification of patients with a low probability of treatment response remains a major clinical challenge. In this context, dynamic monitoring of ENPP1 and IFIT2 may provide complementary host immune information alongside existing response-guided strategies, particularly those based on week 12 HBsAg kinetics. Patients without sustained upregulation of these markers during therapy may have a lower likelihood of favorable response and may benefit from closer on-treatment evaluation and longitudinal monitoring, whereas persistently elevated expression may be associated with a higher probability of favorable on-treatment response during ongoing Peg-IFN-α therapy. Notably, based on the inclusion criteria of the present cohort, the current applicability of this approach is limited to a subgroup of HBeAg-negative CHB patients with low-level viremia, normal or near-normal alanine aminotransferase levels, and predominantly HBV genotype B or C infection. Extrapolation of these findings to broader clinical populations, including HBeAg-positive patients, individuals with different viral genotypes, or those with advanced liver disease, will require further prospective validation. Building upon this dynamic stratification framework, integrating these emerging biomarkers with existing clinical guideline-based algorithms may further optimize patient management in real-world settings. Current EASL and AASLD guidelines emphasize baseline quantitative HBsAg levels and on-treatment HBsAg decline at week 12 as important predictors for Peg-IFN-α response and for guiding treatment continuation decisions (8, 34, 43). However, these virological markers primarily reflect viral antigen dynamics and may not fully capture the complexity of host interferon-mediated immune activation during therapy (16). Our findings suggest that ENPP1 and IFIT2 may provide complementary immunological information that refines this decision-making framework. Specifically, patients who demonstrate a robust early induction of ENPP1 and IFIT2 at week 12, together with a favorable decline in HBsAg, may represent a subgroup more likely to achieve favorable treatment responses during continued Peg-IFN-α therapy. Conversely, patients who fail to exhibit sufficient upregulation of these ISGs, particularly in the presence of suboptimal HBsAg decline, may have a lower probability of achieving a favorable response and may warrant closer on-treatment evaluation and individualized clinical reassessment. Importantly, ENPP1 and IFIT2 should be viewed as complementary host immune indicators that may help refine patient stratification beyond HBsAg-based criteria, thereby supporting a more individualized and biologically informed treatment strategy.

Beyond the performance of the dual-gene models, we further evaluated whether ENPP1 and IFIT2 could provide incremental predictive value when integrated with established HBsAg-related markers. HBsAg and early HBsAg decline are well-established on-treatment indicators for predicting response to pegylated interferon-α therapy in chronic hepatitis B (40, 44). In the present study, the individual discriminative performance of ENPP1 and IFIT2 was generally comparable to that of baseline HBsAg and HBsAg decline at week 12; however, neither marker consistently outperformed these conventional HBsAg-related parameters as a standalone predictor across all evaluated time points. Specifically, ENPP1 measured at weeks 12 and 24 demonstrated predictive performance largely comparable to baseline HBsAg and week 12 HBsAg decline for both VR and SR prediction. In contrast, IFIT2 exhibited stronger predictive capability, particularly at week 24, where it significantly outperformed both baseline HBsAg and week 12 HBsAg decline in predicting VR and SR. These findings suggest that ENPP1 and IFIT2 are not intended to replace established HBsAg-based markers in clinical prediction, but rather provide complementary host immune information that may enhance predictive discrimination during Peg-IFN-α therapy. Importantly, our direct statistical comparisons demonstrated that the predictive value of IFIT2, especially at week 24, extends beyond that of conventional HBsAg-related indicators. The superior predictive performance of IFIT2 at week 24 for both VR and SR suggests that host interferon-stimulated immune responses may capture treatment-related biological changes, thereby providing complementary biological information beyond serum HBsAg kinetics.

Notably, incorporation of ENPP1 and IFIT2 into combined models with baseline HBsAg or HBsAg decline at week 12 significantly improved predictive discrimination compared with conventional HBsAg-related markers alone. For VR prediction, the sequential model integrating ENPP1 at week 12 and IFIT2 at week 24 achieved an AUC of 0.9098, which further increased to 0.9391 after incorporation of baseline HBsAg. Similarly, for SR prediction, the combined model integrating ENPP1 and IFIT2 at week 24 achieved an AUC of 0.9217, increasing to 0.9343 following incorporation of baseline HBsAg. Comparable improvements were also observed when week 12 HBsAg decline was used as the reference marker. Importantly, NRI and IDI analyses consistently confirmed substantial incremental discrimination and reclassification improvement beyond HBsAg-related markers alone. These findings highlight the complementary, rather than substitutive, value of ENPP1 and IFIT2 as host immune biomarkers. Similar patterns have been reported in previous studies, in which the addition of supplementary viral or host biomarkers improved the predictive performance of HBsAg-based models. For example, baseline anti-HBc levels combined with HBsAg enhanced prediction of HBsAg clearance in patients receiving pegylated interferon-α add-on therapy following nucleos(t)ide analogue suppression (17). Likewise, serum HBV RNA measurements have demonstrated incremental predictive value beyond HBsAg decline alone for sustained response and HBsAg loss during pegylated interferon treatment (45). Collectively, these findings suggest that strategies integrating HBsAg-related markers with ENPP1 and IFIT2 expression in PBMCs may provide a more robust framework for guiding individualized early treatment decisions in patients with HBeAg-negative CHB undergoing Peg-IFN-α therapy.

However, several limitations should be acknowledged. First, the majority of enrolled patients were infected with HBV genotypes B or C, and the generalizability of these findings to other genotypes requires further investigation. Second, ENPP1 and IFIT2 mRNA levels were quantified in total PBMCs without further separation of individual immune cell subsets. Because gene expression was measured in the bulk PBMC fraction, we cannot entirely exclude the possibility that subtle changes in the proportions of lymphocytes or monocytes during therapy may have partially influenced the observed differences in mRNA expression levels, although PBMC isolation by density gradient centrifugation (Ficoll) largely removes granulocytes. Given this intrinsic heterogeneity, the observed transcriptional changes cannot be definitively attributed to specific cellular compartments. Therefore, although the dynamic changes observed in bulk PBMCs may reflect alterations in host immune responses during Peg-IFN-α therapy, the absence of subset-specific analyses limits deeper interpretation of the precise cellular mechanisms underlying these associations. Future studies incorporating flow cytometric cell sorting or single-cell transcriptomic approaches may help clarify the cell-specific expression patterns and immunological functions of these biomarkers. Third, emerging viral biomarkers, such as hepatitis B core-related antigen (HBcrAg) and serum HBV RNA (45, 46), which have been reported as valuable on-treatment predictors of Peg-IFN-α response, were not measured in the present cohort. Consequently, direct comparisons between ENPP1/IFIT2-based models and these emerging viral biomarkers could not be performed. Although our analyses demonstrated that ENPP1 and IFIT2 provide incremental predictive value beyond conventional HBsAg-related parameters, whether further integration with HBcrAg or serum HBV RNA could additionally improve predictive performance remains to be determined. Fourth, the follow-up period was relatively limited, precluding assessment of sustained off-treatment responses after therapy cessation. Finally, the prediction models were developed using on-treatment measurements in a relatively small cohort, highlighting the need for validation in larger multicenter populations. Therefore, prospective validation in larger and more diverse cohorts will be essential before these biomarkers can be translated into routine clinical practice.

## Conclusion

This study demonstrates that ENPP1 and IFIT2 mRNA expression in PBMCs correlates closely with virological and serological responses to Peg-IFN-α therapy in HBeAg-negative CHB patients. Establishing these genes as early predictive indicators offers valuable tools for refining clinical decision-making and optimizing therapeutic strategies toward functional cure.

## Data Availability

The raw data supporting the conclusions of this article will be made available by the authors, without undue reservation.
