# Supplementary material for: ENPP1 and IFIT2 in PBMCs as early predictive biomarkers for HBsAg clearance and responses to Peg-IFN-α in HBeAg-negative chronic hepatitis B patients
Source: Front Immunol. 2026 Jun 10;17:1796228. doi: 10.3389/fimmu.2026.1796228 (PMC13290875; doi:10.3389/fimmu.2026.1796228)
Supplement: Supplementary file 11 [file Table1.docx]

| **Table S1** Primer sequences used in this study | |
| --- | --- |
| Primers sequence (5’-3’) | |
| GAPDH RP | 5’-TGACACGTTGGCAGTGG-3’ |
| GAPDH FP | 5’-GGGGCTCTCCAGAACATC-3’ |
| ENPP1 RP | 5’- ATCCTGGCCAGAAAAATGTG-3’ |
| ENPP1 FP | 5’- GAAACGCCTCCTACCCTCTT-3’ |
| IFIT2 RP | 5’- GCTTTCTCCAAGGCTTCTTCAAC-3’ |
| IFIT2 FP | 5’- AGCTACCGTCTGGACAACTG-3’ |
