# Supplementary material for: ENPP1 and IFIT2 in PBMCs as early predictive biomarkers for HBsAg clearance and responses to Peg-IFN-α in HBeAg-negative chronic hepatitis B patients
Source: Front Immunol. 2026 Jun 10;17:1796228. doi: 10.3389/fimmu.2026.1796228 (PMC13290875; doi:10.3389/fimmu.2026.1796228)
Supplement: Supplementary file 12 [file Table2.docx]

| **Table S2** Parameters of the combined predictive models incorporating only ENPP1 and IFIT2. | | | |
| --- | --- | --- | --- |
| Parameter | VR Model (Sequential) |  | SR Model (Dual-marker) |
| Intercept (β₀) | -4.65 (-6.42 to -2.88) |  | -5.18 (-7.35 to -3.01) |
| ENPP1 coefficient (β₁) | 1.59 (0.82-2.36) |  | 1.48 (0.75-2.21) |
| IFIT2 coefficient (β₂) | 2.37 (1.45-3.29) |  | 3.26 (2.08-4.44) |
| ENPP1, Ectonucleotide pyrophosphatase/phosphodiesterase 1; IFIT2, Interferon-induced protein with tetratricopeptide repeats 2; VR, virological response; SR, serological response; The logit function is defined as logit(P) = ln[P/(1-P)], where P is the predicted probability of response. The coefficients (β) shown in the predictive equations were derived from logistic prediction models incorporating only ENPP1 and IFIT2 for prediction purposes. Model coefficients (β₀: intercept; β₁, β₂: regression coefficients for ENPP1 and IFIT2, respectively) were estimated from the training cohort. | | | |
