# Supplementary material for: ENPP1 and IFIT2 in PBMCs as early predictive biomarkers for HBsAg clearance and responses to Peg-IFN-α in HBeAg-negative chronic hepatitis B patients
Source: Front Immunol. 2026 Jun 10;17:1796228. doi: 10.3389/fimmu.2026.1796228 (PMC13290875; doi:10.3389/fimmu.2026.1796228)
Supplement: Supplementary file 13 [file Table3.docx]

| **Table S3** Comparison of baseline characteristics between the training and external validation cohorts. | | | |
| --- | --- | --- | --- |
| Characteristics | Training cohort | External validation cohort | P value |
| Number(n) | 88 | 53 |  |
| Age(year) | 45.00(35.00,51.00) | 46.00(38.50,54.00) | 0.1562 |
| Gender(male/female) | 41/47 | 24/29 |  |
| HBsAg (log10 IU/mL) | 2.846(1.707,3.358) | 2.940(1.675,3.486) | 0.8975 |
| HBV DNA (log10 IU/mL) | 1.699(1.699,3.000) | 1.862(1.699,3.188) | 0.3962 |
| AST(U/L) | 24.00(21.25,31.00) | 26.53±11.48 | 0.1577 |
| ALT(U/L) | 25.50(17.25,39.00) | 28.00(17.00,36.50) | 0.0632 |
| WBC(×10^9/L) | 4.348±1.268 | 4.571±1.190 | 0.1534 |
| PLT(×10^9/L) | 158.0±52.23 | 161.9±47.63 | 0.5361 |
| HBsAg: hepatitis B surface antigen; AST: aspartate aminotransferase; ALT: alanine aminotransferase; WBC: white blood cells; PLT: platelet. The results are presented as the median inter-quartile range or mean ± standard deviation. Bold values are statistically significant P < 0.05. | | | |
