# Supplementary material for: ENPP1 and IFIT2 in PBMCs as early predictive biomarkers for HBsAg clearance and responses to Peg-IFN-α in HBeAg-negative chronic hepatitis B patients
Source: Front Immunol. 2026 Jun 10;17:1796228. doi: 10.3389/fimmu.2026.1796228 (PMC13290875; doi:10.3389/fimmu.2026.1796228)
Supplement: Supplementary file 14 [file Table4.docx]

| **Table S4** Virological response during Peg-IFN-α treatment | | | | |
| --- | --- | --- | --- | --- |
| Virological response (n, %) | 12w | 24w | 36w | 48w |
| HBsAg decreased by >1 log10 IU/ml | 10(11.36%) | 21(23.86%) | 30(34.09%) | 39(44.32%) |
| HBV DNA decreased by >1 log10 IU/ml | 5(5.68%) | 8(9.09%) | 11(12.50%) | 24(27.27%) |
