# Supplementary material for: ENPP1 and IFIT2 in PBMCs as early predictive biomarkers for HBsAg clearance and responses to Peg-IFN-α in HBeAg-negative chronic hepatitis B patients
Source: Front Immunol. 2026 Jun 10;17:1796228. doi: 10.3389/fimmu.2026.1796228 (PMC13290875; doi:10.3389/fimmu.2026.1796228)
Supplement: Supplementary file 15 [file Table5.docx]

| **Table S5** Serological response during Peg-IFN-α treatment | | | | |
| --- | --- | --- | --- | --- |
| Serological response (n, %) | 12w | 24w | 36w | 48w |
| HBsAg clearance | 3(3.41%) | 8(9.09%) | 14(15.91%) | 31(35.23%) |
| HBsAg seroconversion | 0(0.00%) | 6(6.82%) | 10(11.36%) | 18(20.45%) |
