# Supplementary material for: ENPP1 and IFIT2 in PBMCs as early predictive biomarkers for HBsAg clearance and responses to Peg-IFN-α in HBeAg-negative chronic hepatitis B patients
Source: Front Immunol. 2026 Jun 10;17:1796228. doi: 10.3389/fimmu.2026.1796228 (PMC13290875; doi:10.3389/fimmu.2026.1796228)
Supplement: Supplementary file 16 [file Table6.docx]

| **Table S6** Comparison of clinical characteristics between Peg-IFN-α virological response (VR) group and non-virological response (NVR) group | | | | | | | | | | |
| --- | --- | --- | --- | --- | --- | --- | --- | --- | --- | --- |
| characteristics | All | 0w |  | P value | 12w |  | P value | 24w |  | P value |
|  | (n=88) | VR group  (n=39) | NVR group  (n=49) |  | VR group  (n=39) | NVR group  (n=49) |  | VR group  (n=39) | NVR group  (n=49) |  |
| Age(year) | 45.00(35.00  ,51.00) | 45.00(37.00,52.00) | 45.00(35.00  ,50.00) | 0.5206 | 45.00(37.00,52.00) | 45.00(35.00  ,50.00) | 0.5206 | 45.00(37.00,52.00) | 45.00(35.00  ,50.00) | 0.5206 |
| Gender(male/  female) | 41/47 | 19/20 | 22/27 | 0.3301 | 19/20 | 22/27 | 0.3301 | 19/20 | 22/27 | 0.3301 |
| HBV Genotype, n (%) |  |  |  | 0.7628 |  |  | 0.7628 |  |  | 0.7628 |
| Genotype B | 47(53.4) | 21(53.8) | 26(53.1) |  | 21(53.8) | 26(53.1) |  | 21(53.8) | 26(53.1) |  |
| Genotype C | 41(46.6) | 18(46.2) | 23(46.9) |  | 18(46.2) | 23(46.9) |  | 18(46.2) | 23(46.9) |  |
| HBsAg (log10 IU/mL) | 2.846(1.707,  3.358) | 1.693(0.858,2.826) | 3.168(2.745,3.516) | **<0.0001** | 1.225(0.082,2.365) | 2.992(2.584  ,3.425) | **<0.0001** | 0.7210(-0.619,2.066) | 2.958(2.319,3.358) | **<0.0001** |
| HBV DNA (log10 IU/mL) | 1.699(1.699,  3.000) | 1.699(1.699,3.000) | 1.699(1.699,3.195) | 0.6807 | 1.699(1.699,2.000) | 1.699(1.699  ,1.941) | 0.8879 | 1.699(1.699  ,1.699) | 1.699(1.699,1.699) | 0.9275 |
| ALT(U/L) | 25.50(17.25,  39.00) | 26.00(15.00,37.00) | 25.00(18.00,40.00) | 0.7239 | 37.00(23.00,48.00) | 29.00(17.00  ,41.50) | 0.1325 | 37.00(23.00  ,61.00) | 26.00(17.00,45.00) | 0.0598 |
| AST(U/L) | 24.00(21.25,  31.00) | 25.00(22.00,34.00) | 24.00(19.00,30.50) | 0.3914 | 29.00(24.00,41.00) | 27.00(22.50  ,33.50) | 0.2296 | 34.00(25.00  ,43.00) | 27.00(19.00,34.00) | **0.0121** |
| PLT(×10^9/L) | 157.8±49.03 | 153.5±56.23 | 161.7±49.10 | 0.4751 | 138.3±57.29 | 150.3±52.40 | 0.3151 | 115.7±62.48 | 161.6±62.66 | **0.0010** |
| WBC(×10^9/L) | 4.418±1.199 | 4.118±1.229 | 4.531±1.281 | 0.1284 | 4.311±1.356 | 4.456±1.476 | 0.6340 | 3.530(2.580  ,4.140) | 4.270(3.050,5.685) | **0.0117** |
| HBsAg, hepatitis B surface antigen; ALT, alanine aminotransferase; AST: aspartate aminotransferase; WBC: white blood cells; PLT: platelet; VR, virological response; NVR, non-virological response; Bold values are statistically significant P < 0.05. | | | | | | | | | | |
