# Supplementary material for: ENPP1 and IFIT2 in PBMCs as early predictive biomarkers for HBsAg clearance and responses to Peg-IFN-α in HBeAg-negative chronic hepatitis B patients
Source: Front Immunol. 2026 Jun 10;17:1796228. doi: 10.3389/fimmu.2026.1796228 (PMC13290875; doi:10.3389/fimmu.2026.1796228)
Supplement: Supplementary file 17 [file Table7.docx]

| **Table S7** Comparison of clinical characteristics between serological response (SR) group and non-serological (NSR) group | | | | | | | | | | |
| --- | --- | --- | --- | --- | --- | --- | --- | --- | --- | --- |
| characteristics | All | 0w |  | P value | 12w |  | P value | 24w |  | P value |
|  | (n=88) | SR group  (n=31) | NSR group  (n=57) |  | SR group  (n=31) | NSR group  (n=57) |  | SR group  (n=31) | NSR group  (n=57) |  |
| Age(year) | 45.00(35.00  ,51.00) | 45.00(36.00  ,52.00) | 44.00(35.00  ,50.00) | 0.4582 | 45.00(36.00  ,52.00) | 44.00(35.00  ,50.00) | 0.4582 | 45.00(36.00  ,52.00) | 44.00(35.00  ,50.00) | 0.4582 |
| Gender(male/  female) | 41/47 | 14/17 | 27/30 | 0.2561 | 14/17 | 27/30 | 0.2561 | 14/17 | 27/30 | 0.2561 |
| HBV Genotype, n (%) |  |  |  | 0.1123 |  |  | 0.1123 |  |  | 0.1123 |
| Genotype B | 47(53.4) | 19(61.3) | 28(49.1) |  | 19(61.3) | 28(49.1) |  | 19(61.3) | 28(49.1) |  |
| Genotype C | 41(46.6) | 12(38.7) | 29(50.9) |  | 12(38.7) | 29(50.9) |  | 12(38.7) | 29(50.9) |  |
| HBsAg (log10 IU/mL) | 2.846(1.707,3.358) | 1.620(0.687,2.080) | 3.168(2.836,3.516) | **< 0.0001** | 0.653(0.060,  1.678) | 3.024(2.631,3.427) | **< 0.0001** | 0.053(-1.398,1.021) | 2.958(2.289,  3.358) | **< 0.0001** |
| HBV DNA (log10 IU/mL) | 1.699(1.699,3.000) | 1.699(1.699,2.130) | 1.720(1.699,3.004) | 0.0750 | 1.699(1.699,  1.699) | 1.699(1.699,2.000) | 0.1733 | 1.699(1.699,1.699) | 1.699(1.699,  1.699) | 0.1128 |
| ALT(U/L) | 25.50(17.25,39.00) | 24.00(15.00,35.00) | 28.00(18.00,42.00) | 0.4401 | 35.00(17.00,  66.00) | 30.00(20.25,43.00) | 0.4257 | 37.00(25.00,57.00) | 30.00(17.00,  46.00) | 0.1340 |
| AST(U/L) | 24.00(21.25,31.00) | 24.00(22.00,31.00) | 24.00(19.50,31.00) | 0.7924 | 29.00(23.00,  41.00) | 27.50(23.00,35.75) | 0.6479 | 32.00(23.00,43.00) | 27.00(20.00,  38.50) | 0.1569 |
| PLT(×10^9/L) | 157.8±49.03 | 151.4±55.05 | 161.7±50.76 | 0.3932 | 137.8±57.13 | 148.4±53.68 | 0.4046 | 110.0(83.00,151.0) | 154.3±64.83 | **0.0183** |
| WBC(×10^9/L) | 4.418±1.199 | 4.029±1.249 | 4.521±1.255 | 0.0829 | 4.229±1.357 | 4.425(3.045,5.698) | 0.5831 | 3.550(2.580,4.090) | 4.250(3.010,  5.505) | **0.0373** |
| HBsAg, hepatitis B surface antigen; ALT, alanine aminotransferase; AST: aspartate aminotransferase; WBC: white blood cells; PLT: platelet; SR, serological response; NSR, non-serological response; Bold values are statistically significant P < 0.05. | | | | | | | | | | |
