# Supplementary material for: ENPP1 and IFIT2 in PBMCs as early predictive biomarkers for HBsAg clearance and responses to Peg-IFN-α in HBeAg-negative chronic hepatitis B patients
Source: Front Immunol. 2026 Jun 10;17:1796228. doi: 10.3389/fimmu.2026.1796228 (PMC13290875; doi:10.3389/fimmu.2026.1796228)
Supplement: Supplementary file 18 [file Table8.docx]

| **Table S8** On-treatment variables associated with virological response according to univariate and multivariate analyses | | | | | | | | | | | | |
| --- | --- | --- | --- | --- | --- | --- | --- | --- | --- | --- | --- | --- |
| Variables | Univariate analyses |  | Multivariate analyses |  | Univariate analyses |  | Multivariate analyses |  | Univariate analyses |  | Multivariate analyses |  |
|  | OR(95%CI) | P-  value | aOR  (95%CI) | P-value | OR(95%CI) | P-  value | aOR  (95%CI) | P-value | OR(95%CI) | P-  value | aOR  (95%CI) | P-  value |
|  | **Week0** |  |  |  | **Week12** |  |  |  | **Week24** |  |  |  |
| Gender | 1.475(0.458,4.035) | 0.9394 |  |  | 1.475(0.458,4.035) | 0.9394 |  |  | 1.475(0.458,4.035) | 0.9394 |  |  |
| Age | 1.012(0.969,1.056) | 0.5741 |  |  | 1.012(0.969,1.056) | 0.5741 |  |  | 1.012(0.969,1.056) | 0.5741 |  |  |
| HBsAg | 0.281(0.158,0.501) | **<0.0001** | 0.118(0.045,0.309) | **<0.0001** | 0.329(0.202,0.536) | **<0.0001** | 0.343(0.200  ,0.587) | **0.0001** | 0.322(0.199,0.520) | **<0.0001** | 0.455(0.244  ,0.851) | **0.0137** |
| HBV DNA | 1.118(0.579,2.159) | 0.7389 |  |  | 1.189(0.410,3.446) | 0.7485 |  |  | 0.968(0.110,8.482) | 0.9767 |  |  |
| ALT | 1.006(0.993,1.019) | 0.3285 |  |  | 1.018(0.999,1.037) | 0.0611 |  |  | 1.017(1.000,1.034) | **0.0386** |  |  |
| AST | 1.007(0.983,1.032) | 0.5454 |  |  | 1.002(0.984,1.020) | 0.7849 |  |  | 1.022(0.998,1.048) | 0.0682 |  |  |
| WBC | 0.767(0.544,1.081) | 0.1308 |  |  | 0.929(0.688,1.254) | 0.6330 |  |  | 0.788(0.608,1.022) | 0.0729 |  |  |
| PLT | 0.996(0.988,1.005) | 0.4636 |  |  | 0.995(0.988,1.003) | 0.3069 |  |  | 0.987(0.980,0.995) | **0.0022** |  |  |
| HBV Genotype (B vs. C) | 0.765(0.456, 1.368) | 0.0893 |  |  | 0.765(0.456, 1.368) | 0.0893 |  |  | 0.765(0.456, 1.368) | 0.0893 |  |  |
| ENPP1 | 1.194(0.694,2.055) | 0.5213 |  |  | 1.720(1.198,2.470) | **0.0032** | 1.805(1.192  ,2.764) | **0.0092** | 1.432(1.179,1.740) | **0.0002** | 1.560(1.201  ,1.975) | **0.0026** |
| IFIT2 | 0.995(0.682,1.453) | 0.9826 |  |  | 1.599(1.116,2.291) | **0.0104** | 1.766(1.131  ,2.537) | **0.0119** | 2.944(1.882,4.604) | **0.0001** | 2.394(1.598  ,4.306) | **0.0011** |
| Values expressed as odds ratio (OR) and 95% confidence interval (CI). aOR, adjusted odds ratio; ENPP1, ectonucleotide pyrophosphatase/phosphodiesterase 1; IFIT2, interferon-induced protein with tetratricopeptide repeats 2; HBsAg, hepatitis B surface antigen; ALT, alanine aminotransferase; AST, aspartate aminotransferase; WBC, white blood cells; PLT: platelet. For brevity, only variables retained in the final stepwise multivariable model are displayed in the multivariate columns, whereas all clinically relevant candidate variables, including HBV genotype, were entered into the multivariable selection procedure. Bold values are statistically significant P < 0.05. | | | | | | | | | | | | |
