# Supplementary material for: ENPP1 and IFIT2 in PBMCs as early predictive biomarkers for HBsAg clearance and responses to Peg-IFN-α in HBeAg-negative chronic hepatitis B patients
Source: Front Immunol. 2026 Jun 10;17:1796228. doi: 10.3389/fimmu.2026.1796228 (PMC13290875; doi:10.3389/fimmu.2026.1796228)
Supplement: Supplementary file 20 [file Table10.docx]

| **Table S10** The predictive performance of ENPP1 and IFIT2 mRNA levels in predicting VR and SR to 48 weeks of Peg-IFN-α treatment. | | | | | | | | | |
| --- | --- | --- | --- | --- | --- | --- | --- | --- | --- |
|  |  | ENPP1 | IFIT2 |  | ENPP1 | IFIT2 |  | ENPP1 | IFIT2 |
| VR prediction |  |  | **Week 0** |  |  | **Week12** |  |  | **Week24** |
|  | AUC | 0.5055 | 0.5024 |  | 0.7645 | 0.7080 |  | 0.7298 | 0.8791 |
|  | (95% CI) | (0.3795 - 0.6315) | (0.3801 - 0.6247) |  | (0.6640 - 0.8651) | (0.6002 - 0.8158) |  | (0.6631 - 0.8660) | (0.8352 - 0.9597) |
|  | Cut-off value | 0.9096 | 1.1713 |  | 1.7789 | 0.8548 |  | 2.3194 | 2.9699 |
|  | Sensitivity (%) | 48.70 | 66.70 |  | 64.10 | 87.20 |  | 76.90 | 82.10 |
|  | Specificity (%) | 67.30 | 44.90 |  | 85.70 | 49.00 |  | 69.40 | 95.90 |
|  | P value | 0.9297 | 0.9698 |  | **< 0.0001** | **0.0008** |  | **< 0.0001** | **< 0.0001** |
| SR prediction |  |  | **Week 0** |  |  | **Week12** |  |  | **Week24** |
|  | AUC | 0.5241 | 0.5093 |  | 0.7102 | 0.8223 |  | 0.7399 | 0.8879 |
|  | (95% CI) | (0.3956 - 0.6525) | (0.3812 - 0.6375) |  | \| (0.6054 - 0.8310) \| \| --- \| | (0.7352 - 0.9094) |  | (0.6046 - 0.8351) | (0.8495 - 0.9796) |
|  | Cut-off value | 0.8621 | 1.0369 |  | 1.4772 | 2.2787 |  | 3.3767 | 3.4042 |
|  | Sensitivity (%) | 48.40 | 64.50 |  | 64.50 | 61.30 |  | 61.40 | 93.50 |
|  | Specificity (%) | 68.40 | 49.10 |  | 70.20 | 87.70 |  | 77.20 | 96.50 |
|  | P value | 0.7105 | 0.8854 |  | **0.0008** | **< 0.0001** |  | **0.0007** | **< 0.0001** |
| ENPP1, ectonucleotide pyrophosphatase/phosphodiesterase 1; IFIT2, interferon-induced protein with tetratricopeptide repeats 2; AUC, area under ROC curve; CI, confidence interval; VR, virological response; SR, serological response; Bold values are statistically significant P < 0.05. | | | | | | | | | |
