# Supplementary material for: ENPP1 and IFIT2 in PBMCs as early predictive biomarkers for HBsAg clearance and responses to Peg-IFN-α in HBeAg-negative chronic hepatitis B patients
Source: Front Immunol. 2026 Jun 10;17:1796228. doi: 10.3389/fimmu.2026.1796228 (PMC13290875; doi:10.3389/fimmu.2026.1796228)
Supplement: Supplementary file 21 [file Table11.docx]

| **Table S11** The combined predictive performance of ENPP1 and IFIT2 mRNA levels for predicting VR and SR after 48 weeks of Peg-IFN-α treatment. | | | |
| --- | --- | --- | --- |
|  | VR prediction |  | SR prediction |
| Predictors | ENPP1 (Week 12) + IFIT2 (Week 24) |  | ENPP1 (Week 24) + IFIT2 (Week 24) |
| AUC | 0.9098 |  | 0.9217 |
| (95% CI) | (0.8746 - 0.9725) |  | (0.8834 - 0.9907) |
| Cut-off value | 0.2303 |  | 0.8645 |
| Sensitivity (%) | 92.30 |  | 87.10 |
| Specificity (%) | 83.70 |  | 98.20 |
| P value | **< 0.0001** |  | **< 0.0001** |
| ENPP1, Ectonucleotide pyrophosphatase/phosphodiesterase 1; IFIT2, Interferon-induced protein with tetratricopeptide repeats 2; AUC, area under ROC curve; CI, confidence interval; VR, virological response; SR, serological response; Bold values are statistically significant P < 0.05. | | | |
