# Supplementary material for: ENPP1 and IFIT2 in PBMCs as early predictive biomarkers for HBsAg clearance and responses to Peg-IFN-α in HBeAg-negative chronic hepatitis B patients
Source: Front Immunol. 2026 Jun 10;17:1796228. doi: 10.3389/fimmu.2026.1796228 (PMC13290875; doi:10.3389/fimmu.2026.1796228)
Supplement: Supplementary file 22 [file Table12.docx]

| **Table S12** Predictive performance of ENPP1, IFIT2, and the combined model for VR and SR in the external validation cohort. | | | |
| --- | --- | --- | --- |
|  | VR prediction |  | SR prediction |
| Predictors | ENPP1 (Week 12) + IFIT2 (Week 24) |  | ENPP1 (Week 24) + IFIT2 (Week 24) |
| AUC | 0.8818 |  | 0.8974 |
| (95% CI) | (0.7918 - 0.9718) |  | (0.8146 - 0.9803) |
| Cut-off value | 0.2303 |  | 0.8645 |
| Sensitivity (%) | 81.30 |  | 78.57 |
| Specificity (%) | 84.85 |  | 82.05 |
| P value | **< 0.0001** |  | **< 0.0001** |
| ENPP1, Ectonucleotide pyrophosphatase/phosphodiesterase 1; IFIT2, Interferon-induced protein with tetratricopeptide repeats 2; AUC, area under ROC curve; CI, confidence interval; VR, virological response; SR, serological response; The cut-off values applied in this external validation cohort were predefined and strictly derived from the primary training cohort; Bold values are statistically significant P < 0.05. | | | |
