# Supplementary material for: ENPP1 and IFIT2 in PBMCs as early predictive biomarkers for HBsAg clearance and responses to Peg-IFN-α in HBeAg-negative chronic hepatitis B patients
Source: Front Immunol. 2026 Jun 10;17:1796228. doi: 10.3389/fimmu.2026.1796228 (PMC13290875; doi:10.3389/fimmu.2026.1796228)
Supplement: Supplementary file 23 [file Table13.docx]

| **Table S13** Calibration performance in the external validation cohort. | | | |
| --- | --- | --- | --- |
| Predictive Model | Intercept | Slope | Brier Score |
| VR | 0.016 | 1.091 | 0.1386 |
| SR | 0.031 | 1.028 | 0.1161 |
| VR, virological response; SR, serological response. | | | |
