# Supplementary material for: ENPP1 and IFIT2 in PBMCs as early predictive biomarkers for HBsAg clearance and responses to Peg-IFN-α in HBeAg-negative chronic hepatitis B patients
Source: Front Immunol. 2026 Jun 10;17:1796228. doi: 10.3389/fimmu.2026.1796228 (PMC13290875; doi:10.3389/fimmu.2026.1796228)
Supplement: Supplementary file 24 [file Table14.docx]

| **Table S14** Model performance in the internal validation (bootstrap resampling). | | | |
| --- | --- | --- | --- |
| Predictive Model | Optimism Corrected C-index | Intercept | Brier scores |
| VR | 0.8823 | 0.0026 | 0.1125 |
| SR | 0.9054 | 0.0031 | 0.1078 |
| VR, virological response; SR, serological response. | | | |
