# Supplementary material for: ENPP1 and IFIT2 in PBMCs as early predictive biomarkers for HBsAg clearance and responses to Peg-IFN-α in HBeAg-negative chronic hepatitis B patients
Source: Front Immunol. 2026 Jun 10;17:1796228. doi: 10.3389/fimmu.2026.1796228 (PMC13290875; doi:10.3389/fimmu.2026.1796228)
Supplement: Supplementary file 25 [file Table15.docx]

| **Table S15** Comparison of predictive performance of ENPP1 and IFIT2 at weeks 12 and 24 with baseline HBsAg for predicting VR and SR. | | | | | | | | |
| --- | --- | --- | --- | --- | --- | --- | --- | --- |
| Response prediction |  | ENPP1(week 12) | IFIT2 (week 12) | HBsAg (baseline) |  | ENPP1(week 24) | IFIT2 (week 24) | HBsAg (baseline) |
| VR prediction | AUC | 0.7645 | 0.7080 | 0.7393 |  | 0.7298 | 0.8791 | 0.7393 |
|  | (95% CI) | (0.6640 - 0.8651) | (0.6002 - 0.8158) | (0.6311 - 0.8475) |  | (0.6631 - 0.8660) | (0.8352 - 0.9597) | (0.6311 - 0.8475) |
|  | Cut-off value | 1.7789 | 0.8548 | 1.7253 |  | 2.3194 | 2.9699 | 1.7253 |
|  | Sensitivity (%) | 64.10 | 87.20 | 58.75 |  | 76.90 | 82.10 | 58.75 |
|  | Specificity (%) | 85.70 | 49.00 | 83.70 |  | 69.40 | 95.90 | 83.70 |
|  | P value (DeLong's test, vs. baseline HBsAg) | 0.6531 | 0.8492 |  |  | 0.5359 | **0.0103** |  |
| SR prediction | AUC | 0.7102 | 0.8223 | 0.7266 |  | 0.7399 | 0.8879 | 0.7266 |
|  | (95% CI) | (0.6054 - 0.8310) | (0.7352 - 0.9094) | (0.6071 - 0.8461) |  | (0.6046 - 0.8351) | (0.8495 - 0.9796) | (0.6071 - 0.8461) |
|  | Cut-off value | 1.4772 | 2.2787 | 1.9595 |  | 3.3767 | 3.4042 | 1.9595 |
|  | Sensitivity (%) | 64.50 | 61.30 | 53.30 |  | 61.40 | 93.50 | 53.30 |
|  | Specificity (%) | 70.20 | 87.70 | 87.90 |  | 77.20 | 96.50 | 87.90 |
|  | P value (DeLong's test, vs. baseline HBsAg) | 0.7652 | **0.0138** |  |  | 0.9634 | **0.0078** |  |
| HBsAg, hepatitis B surface antigen; ENPP1, ectonucleotide pyrophosphatase/phosphodiesterase 1; IFIT2, interferon-induced protein with tetratricopeptide repeats 2; AUC, area under ROC curve; CI, confidence interval; VR, virological response; SR, serological response; Bold values are statistically significant P < 0.05. | | | | | | | | |

| **Table S16** Comparison of predictive performance of ENPP1 and IFIT2 at weeks 12 and 24 with HBsAg decline at week 12 for predicting VR and SR. | | | | | | | | |
| --- | --- | --- | --- | --- | --- | --- | --- | --- |
| Response prediction |  | ENPP1 (week 12) | IFIT2 (week 12) | HBsAg decline (week 12) |  | ENPP1 (week 24) | IFIT2 (week 24) | HBsAg decline (week 12) |
| VR prediction | AUC | 0.7645 | 0.7080 | 0.7758 |  | 0.7298 | 0.8791 | 0.7758 |
|  | (95% CI) | (0.6640 - 0.8651) | (0.6002 - 0.8158) | (0.6781 - 0.8734) |  | (0.6631 - 0.8660) | (0.8352 - 0.9597) | (0.6781 - 0.8734) |
|  | Cut-off value | 1.7789 | 0.8548 | 0.4536 |  | 2.3194 | 2.9699 | 0.4536 |
|  | Sensitivity (%) | 64.10 | 87.20 | 56.40 |  | 76.90 | 82.10 | 56.40 |
|  | Specificity (%) | 85.70 | 49.00 | 87.80 |  | 69.40 | 95.90 | 87.80 |
|  | P value (DeLong's test, vs. HBsAg decline at week 12) | 0.7592 | **0.0453** |  |  | 0.4539 | **0.0325** |  |
| SR prediction | AUC | 0.7102 | 0.8223 | 0.8270 |  | 0.7399 | 0.8879 | 0.8270 |
|  | (95% CI) | (0.6054 - 0.8310) | (0.7352 - 0.9094) | (0.7267 - 0.9273) |  | (0.6046 - 0.8351) | (0.8495 - 0.9796) | (0.7267 - 0.9273) |
|  | Cut-off value | 1.4772 | 2.2787 | 0.8751 |  | 3.3767 | 3.4042 | 0.8751 |
|  | Sensitivity (%) | 64.50 | 61.30 | 77.10 |  | 61.40 | 93.50 | 77.10 |
|  | Specificity (%) | 70.20 | 87.70 | 86.50 |  | 77.20 | 96.50 | 86.50 |
|  | P value (DeLong's test, vs. HBsAg decline at week 12) | **0.0268** | 0.2960 |  |  | **0.0486** | **0.0375** |  |
| HBsAg, hepatitis B surface antigen; ENPP1, ectonucleotide pyrophosphatase/phosphodiesterase 1; IFIT2, interferon-induced protein with tetratricopeptide repeats 2; AUC, area under ROC curve; CI, confidence interval; VR, virological response; SR, serological response; Bold values are statistically significant P < 0.05. | | | | | | | | |

| **Table S17** Comparison of predictive performance between baseline HBsAg and combined models for predicting VR and SR. | | | | |
| --- | --- | --- | --- | --- |
| Response  prediction |  | HBsAg (baseline) | ENPP1 (week 12) + IFIT2 (week 24) | ENPP1 (week 12) + IFIT2 (week 24) + HBsAg (baseline) |
| VR  prediction | AUC | 0.7393 | 0.9098 | 0.9391 |
|  | (95% CI) | (0.6311 - 0.8475) | (0.8746 - 0.9725) | (0.8887 - 0.9895) |
|  | Cut-off value | 1.7253 | 0.2303 | 0.5574 |
|  | Sensitivity (%) | 58.75 | 92.30 | 84.60 |
|  | Specificity (%) | 83.70 | 83.70 | 91.80 |
|  | P value (DeLong's test, vs. baseline HBsAg) |  | **0.0076** | **0.0095** |
|  |  | HBsAg (baseline) | ENPP1 (week 24) + IFIT2 (week 24) | ENPP1 (week 24) + IFIT2 (week 24) + HBsAg (baseline) |
| SR  prediction | AUC | 0.7266 | 0.9217 | 0.9343 |
|  | (95% CI) | (0.6071 - 0.8461) | (0.8834 - 0.9907) | (0.8847 - 0.9840) |
|  | Cut-off value | 1.9595 | 0.8645 | 0.3655 |
|  | Sensitivity (%) | 53.30 | 87.10 | 87.10 |
|  | Specificity (%) | 87.90 | 98.20 | 86.00 |
|  | P value (DeLong's test, vs. baseline HBsAg) |  | **0.0155** | **0.0076** |
| HBsAg, hepatitis B surface antigen; ENPP1, ectonucleotide pyrophosphatase/phosphodiesterase 1; IFIT2, interferon-induced protein with tetratricopeptide repeats 2; AUC, area under ROC curve; CI, confidence interval; VR, virological response; SR, serological response; Bold values are statistically significant P < 0.05. | | | | |

| **Table S18** Incremental predictive value of the integrated models compared with baseline HBsAg alone. | | | | | |
| --- | --- | --- | --- | --- | --- |
| Clinical Endpoint | Comparison | Continuous NRI (95% CI) | P value | IDI (95% CI) | P value |
| VR | Integrated model vs. Baseline HBsAg | 0.948 (0.563-1.333) | **< 0.0001** | 0.241 (0.156-0.326) | **< 0.0001** |
| SR | Integrated model vs. Baseline HBsAg | 1.072 (0.648-1.496) | **0.0019** | 0.279 (0.184-0.374) | **0.0012** |
| VR, virological response; SR, serological response; NRI, net reclassification improvement; IDI, integrated discrimination improvement; CI, confidence interval. The integrated model for VR/SR incorporates ENPP1 (week 12), IFIT2 (week 24), and baseline HBsAg; Bold values are statistically significant P < 0.05. | | | | | |

| **Table S19** Comparison of predictive performance between baseline HBsAg and combined models for predicting VR and SR. | | | | |
| --- | --- | --- | --- | --- |
| Response  prediction |  | HBsAg decline (week 12) | ENPP1 (week 12) + IFIT2 (week 24) | ENPP1 (week 12) + IFIT2 (week 24) + HBsAg decline (week 12) |
| VR  prediction | AUC | 0.7758 | 0.9098 | 0.9214 |
|  | (95% CI) | (0.6781 - 0.8734) | (0.8746 - 0.9725) | (0.8770 - 0.9859) |
|  | Cut-off value | 0.4536 | 0.2303 | 0.2812 |
|  | Sensitivity (%) | 56.40 | 92.30 | 92.30 |
|  | Specificity (%) | 87.80 | 83.70 | 85.70 |
|  | P value (DeLong's test, vs. week 12 HBsAg decline) |  | **0.0205** | **0.0149** |
|  |  | HBsAg decline (week 12) | ENPP1 (week 24) + IFIT2 (week 24) | ENPP1 (week 24) + IFIT2 (week 24) + HBsAg decline (week 12) |
| SR  prediction | AUC | 0.8270 | 0.9217 | 0.9315 |
|  | (95% CI) | (0.7267 - 0.9273) | (0.8834 - 0.9907) | (0.8685 - 0.9945) |
|  | Cut-off value | 0.8751 | 0.8645 | 0.5772 |
|  | Sensitivity (%) | 77.10 | 87.10 | 80.60 |
|  | Specificity (%) | 86.50 | 98.20 | 91.60 |
|  | P value (DeLong's test, vs. week 12 HBsAg decline) |  | **0.0236** | **0.0152** |
| HBsAg, hepatitis B surface antigen; ENPP1, ectonucleotide pyrophosphatase/phosphodiesterase 1; IFIT2, interferon-induced protein with tetratricopeptide repeats 2; AUC, area under ROC curve; CI, confidence interval; VR, virological response; SR, serological response; Bold values are statistically significant P < 0.05. | | | | |

| **Table S20** Incremental predictive value of the integrated models compared with HBsAg decline at week 12 alone. | | | | | |
| --- | --- | --- | --- | --- | --- |
| Clinical Endpoint | Comparison | Continuous NRI (95% CI) | P value | IDI (95% CI) | P value |
| VR | Integrated model vs. Week 12 HBsAg decline | 0.603 (0.281-0.925) | **0.0026** | 0.138 (0.071-0.205) | **0.0037** |
| SR | Integrated model vs. Week 12 HBsAg decline | 0.572 (0.231-0.913) | **0.0045** | 0.119 (0.057-0.181) | **0.0061** |
| VR, virological response; SR, serological response; NRI, net reclassification improvement; IDI, integrated discrimination improvement; CI, confidence interval. The integrated model for VR/SR incorporates ENPP1 (week 12), IFIT2 (week 24), and week 12 HBsAg decline ; Bold values are statistically significant P < 0.05. | | | | | |
