# Supplementary material for: ENPP1 and IFIT2 in PBMCs as early predictive biomarkers for HBsAg clearance and responses to Peg-IFN-α in HBeAg-negative chronic hepatitis B patients
Source: Front Immunol. 2026 Jun 10;17:1796228. doi: 10.3389/fimmu.2026.1796228 (PMC13290875; doi:10.3389/fimmu.2026.1796228)
Supplement: Supplementary file 26 [file DataSheet1.pdf]

(1) Peripheral blood collection and PBMC extraction

Peripheral venous blood samples were collected in EDTA-anticoagulated vacuum tubes.

Each sample volume was 4 mL, which was equally divided into two tubes.

Samples were kept at 4°C immediately after collection and processed within 2 hours.

Plasma separation

All procedures were performed under sterile conditions. The biosafety cabinet was disinfected by ultraviolet irradiation for 30 minutes prior to sample handling.

During this period, blood samples were centrifuged at 4°C and 1200 rpm for 10 minutes for plasma separation.

Following centrifugation, tubes were opened inside the biosafety cabinet, and the supernatant was carefully aspirated.

The collected supernatant was centrifuged again at 4°C and 3000 rpm for 15 minutes to remove residual cellular components.

The extracted supernatant was collected and transferred to sterilized EP tubes, with 500 μL aliquots in each tube, which were then stored at −80°C for future analysis.

PBMC isolation

Whole blood was diluted 1:1 with 1× PBS, gently mixed, and divided into two tubes.

Ficoll lymphocyte separation medium (4 mL) was carefully layered beneath the diluted blood using a capillary pipette.

The sample was centrifuged at 400 g for 40 minutes at 20–24°C.

After centrifugation, the mononuclear cell layer at the plasma–Ficoll interface was carefully collected and transferred to a new EP tube.

The procedure was repeated when necessary to increase PBMC yield.

PBMCs were resuspended in cryopreservation medium consisting of RPMI 1640 supplemented with 10% fetal bovine serum and 50% 2× serum-free freezing solution.

After cell counting, samples containing more than 3×10^6 cells were aliquoted into 1.5 mL cryovials.

The cryovials were placed in a gradient freezing box and stored at −80°C for freezing. After 24 hours, they were transferred to a liquid nitrogen tank for long-term storage to maintain cell viability.

(2) Extraction of total RNA from cells

Total RNA was extracted using TRIzol reagent according to standard protocols, and all procedures were carried out in an RNase-free biosafety cabinet.

The centrifuge was precooled to 4°C before RNA extraction.

After removal of the culture medium, cells were washed twice with PBS, and residual buffer was aspirated.

TRIzol reagent (1 mL per well) was added to lyse the cells, followed by

incubation at room temperature for 3–5 minutes.

The lysate was transferred to a 1.5 mL RNase-free EP tube, mixed thoroughly, and incubated at room temperature for 5 minutes.

Chloroform was added, and the tubes were gently inverted to mix. After incubation at room temperature, phase separation was achieved by centrifugation.

Centrifuge the mixture at 4°C, with a speed of 12,000 g for 15 minutes.

The upper aqueous phase was carefully transferred to a new RNase-free EP tube, and RNA was precipitated by the addition of isopropanol followed by incubation at room temperature for 10 minutes.

Samples were centrifuged at 4°C and 12,000 g for 10 minutes.

The supernatant was discarded, and the RNA pellet was washed with 75% ethanol.

After centrifugation at 4°C and 7,500 g for 5 minutes, the ethanol was removed, and the pellet was air-dried.

RNA was dissolved in 10–20 µL of DEPC-treated water and stored at 4°C for subsequent analysis.

(3) Reverse Transcription of RNA to cDNA

According to the instructions of the PrimeScript™ RT Reagent Kit with gDNA Eraser, perform the following steps:

a. To remove genomic DNA contamination, reaction mixtures were prepared on ice in accordance with the manufacturer-recommended

component ratios. To ensure sufficient reagents and experimental consistency, reaction mixtures were prepared at twice the volume required for the total number of reactions.

Reagent	Dosage
5×gDNA Eraser Buffer	2.0ul
Total RNA	1ug
Rnase Tree ddH_20	add to 10ul
gDNA Eraser	1.0ul

b. The reaction mixtures were kept at room temperature for 5–10 minutes before use.

c. Reverse transcription reactions were assembled on ice. To ensure the accuracy and stability of the reaction, the reagents should be pre-prepared in an amount that is 2 times the required number of reactions. A volume of 10 μL of the reaction mixture was added to each tube and gently mixed prior to incubation.

Reagent	Dosage
Reaction solution of step a	10.0ul
PrimeScript RT Enzyme Mix I	1.0ul
5×PrimeScript Buffer II	4.0ul
Rnase Tree ddH_20	4.0ul
RT Prime Mix	1.0ul

d. The reverse transcription reaction should be performed in a PCR

machine with the following conditions: 37°C for 15 minutes → 85°C for 5 seconds → 4°C for 1 minute.

e. Store the cDNA at -20°C.

(4) Amplifying the target gene

Follow the instructions of the SYBR Green qPCR Master Mix kit. Perform the entire process on ice, and prepare the reaction mixture according to the following components:

Reagent	Dosage
SYBR Green	5.0ul
FP（10uM）	0.2ul
RP（10uM）	0.2ul
cDNA	1.0ul
ddH_20	3.6ul

Use the Bio-Rad fluorescence quantitative PCR instrument CFX96 to perform real-time fluorescence quantitative PCR on cDNA with the following conditions:

- Pre-denaturation: 95°C for 30 seconds
- PCR: 95°C for 5 seconds; 60°C for 30 seconds × 40 cycles
- Melting: 95°C for 5 seconds; 60°C for 1 minute; 95°C
- Cooling: 50°C for 30 seconds
- Read fluorescence signal.
